# What we mean when we say semantic: Toward a multidisciplinary semantic glossary

**DOI:** 10.3758/s13423-024-02556-7

**Published:** 2024-09-04

**Authors:** Jamie Reilly, Cory Shain, Valentina Borghesani, Philipp Kuhnke, Gabriella Vigliocco, Jonathan E. Peelle, Bradford Z. Mahon, Laurel J. Buxbaum, Asifa Majid, Marc Brysbaert, Anna M. Borghi, Simon De Deyne, Guy Dove, Liuba Papeo, Penny M. Pexman, David Poeppel, Gary Lupyan, Paulo Boggio, Gregory Hickok, Laura Gwilliams, Leonardo Fernandino, Daniel Mirman, Evangelia G. Chrysikou, Chaleece W. Sandberg, Sebastian J. Crutch, Liina Pylkkänen, Eiling Yee, Rebecca L. Jackson, Jennifer M. Rodd, Marina Bedny, Louise Connell, Markus Kiefer, David Kemmerer, Greig de Zubicaray, Elizabeth Jefferies, Dermot Lynott, Cynthia S.Q. Siew, Rutvik H. Desai, Ken McRae, Michele T. Diaz, Marianna Bolognesi, Evelina Fedorenko, Swathi Kiran, Maria Montefinese, Jeffrey R. Binder, Melvin J. Yap, Gesa Hartwigsen, Jessica Cantlon, Yanchao Bi, Paul Hoffman, Frank E. Garcea, David Vinson

**Affiliations:** 1 Temple University, Philadelphia, PA USA; 2 Massachusetts Institute of Technology, Cambridge, MA USA; 3 University of Geneva, Geneva, Switzerland; 4 Max Planck Institute for Human Cognitive and Brain Sciences, Leipzig, Germany; 5 University College London, London, UK; 6 Northeastern University, Boston, MA USA; 7 Carnegie Mellon University, Pittsburgh, PA USA; 8 Thomas Jefferson University, Moss Rehabilitation Research Institute, Elkins Park, PA USA; 9 University of Oxford, Oxford, UK; 10 University of Ghent, Ghent, Belgium; 11 Sapienza University of Rome, Rome, Italy; 12 University of Melbourne, Melbourne, Australia; 13 University of Louisville, Louisville, KY USA; 14 Centre National de La Recherche Scientifique (CNRS), University Claude-Bernard Lyon, Lyon, France; 15 Western University, London, ON Canada; 16 New York University, New York, NY USA; 17 University of Wisconsin, Madison, WI USA; 18 Universidade Presbiteriana Mackenzie, São Paulo, Brazil; 19 University of California at Irvine, Irvine, CA USA; 20 Stanford University, Stanford, CA USA; 21 Medical College of Wisconsin, Milwaukee, WI USA; 22 University of Edinburgh, Edinburgh, UK; 23 Drexel University, Philadelphia, PA USA; 24 Pennsylvania State University, State College, PA USA; 25 University College London, London, UK; 26 University of Connecticut, Storrs, CT USA; 27 University of York, York, UK; 28 Johns Hopkins University, Baltimore, MD USA; 29 Maynooth University, Maynooth, Ireland; 30 Ulm University, Ulm, Germany; 31 Purdue University, West Lafayette, IN USA; 32 Queensland University of Technology, Brisbane, QLD Australia; 33 National University of Singapore, Singapore, Singapore; 34 University of South Carolina, Columbia, SC USA; 35 University of Bologna, Bologna, Italy; 36 Boston University, Boston, MA USA; 37 University of Padua, Padua, Italy; 38 National University of Singapore, Singapore, Singapore; 39 Beijing Normal University, Beijing, China; 40 University of Rochester, Rochester, NY USA; 41 Leipzig University, Leipzig, Germany

**Keywords:** Semantic memory, Concept, Representation, Concreteness, Abstraction, Lexical

## Abstract

Tulving characterized semantic memory as a vast repository of meaning that underlies language and many other cognitive processes. This perspective on lexical and conceptual knowledge galvanized a new era of research undertaken by numerous fields, each with their own idiosyncratic methods and terminology. For example, “concept” has different meanings in philosophy, linguistics, and psychology. As such, many fundamental constructs used to delineate semantic theories remain underspecified and/or opaque. Weak construct specificity is among the leading causes of the replication crisis now facing psychology and related fields. Term ambiguity hinders cross-disciplinary communication, falsifiability, and incremental theory-building. Numerous cognitive subdisciplines (e.g., vision, affective neuroscience) have recently addressed these limitations via the development of consensus-based guidelines and definitions. The project to follow represents our effort to produce a multidisciplinary semantic glossary consisting of succinct definitions, background, principled dissenting views, ratings of agreement, and subjective confidence for 17 target constructs (e.g., abstractness, abstraction, concreteness, concept, embodied cognition, event semantics, lexical-semantic, modality, representation, semantic control, semantic feature, simulation, semantic distance, semantic dimension)*.* We discuss potential benefits and pitfalls (e.g., implicit bias, prescriptiveness) of these efforts to specify a common nomenclature that other researchers might index in specifying their own theoretical perspectives (e.g., They said X, but I mean Y).

## Introduction

Scientific discovery is not immune to cognitive biases that influence human reasoning. People tend to hold strong convictions about the veracity of their own beliefs (Pulford et al., 2018; Ross & Ward, 1996), often overestimating their understanding of complex phenomena (Dunning, 2011). Such biases threaten precision, generalizability, and reproducibility of empirical measurement. Moreover, complex constructs such as “dark matter” and “happiness” often resist straightforward explanations. The hard work of formal theory-building involves defining core terms. When fixed reference points do not exist, it is impossible to falsify predictions, calibrate disparate viewpoints, and assess the validity of empirical measures (Flake & Fried, 2020). As such, limited construct specificity has been identified as one of the leading causes of the ongoing replication crisis in psychology and related fields (Korbmacher et al., 2023; Oberauer & Lewandowsky, 2019).

In response, numerous workgroups have recently published consensus definitions, practice, and/or standardization guidelines for domains such as visual attention (Liesefeld et al., 2024), mental state attribution (Quesque et al., 2024), cerebellum and social cognition (Van Overwalle et al., 2020), cerebellum and language (Mariën et al., 2014), cognitive performance under pressure (Albertella et al., 2023), cognitive frailty (Kelaiditi et al., 2013), biomarker-based diagnosis of Alzheimer’s disease (Frisoni et al., 2017), and bilingual aphasia assessment (Martínez-Ferreiro et al., 2024).

## Goals of the current workgroup

Much of the core lexicon used to describe semantic phenomena is opaque, ambiguous, or only accessible to a narrow range of experts (see Calzavarini, 2023). The evolution of a narrow vernacular is antithetical to the interdiscplinary promise of cognitive science. As scientists who specialize in the study of semantics, many of us have struggled to understand exactly what people mean when they say that a concept is amodal or that a word is abstract. For example, Machery (2009) has argued that ambiguity and misinterpretation regarding *concept* is so ubiquitous that “use of the term ‘concept’ may damage our psychological theorizing” (p. 245). An elimintativist perspective would involve shunning the use of such terms (Raffman, 2010).

We convened a multidisplinary workgroup in an attempt to reconcile points of convergence/divergence, and produce an semantic glossary that other researchers might find useful in disambiguating or align their own perspectives against (e.g., They said X, but I mean Y).

We developed this glossary with attention to several additional constraints, including multidiciplinary accessibility (i.e., definitions should be accessible to nonexperts and provide supporting didactic background) and mechanism(s) for expressing principled disagreements with the majority definition.

## What are the benefits of a semantic glossary?

Although the study of concepts can be traced back thousands of years, many researchers link the modern era of psychological semantic research to Endel Tulving’s (1927–2023) seminal book chapter, “Episodic and Semantic Memory” (1972).^1^ The post-Tulving era of semantic research has since been undertaken by numerous disciplines, each with its own idiosyncratic lexicon, theories, and methods. For example, terms such as *concept* and *amodal* have fundamentally different meanings between philosophers, linguists, and cognitive neuroscientists (for discussion and historical perspectives, see Calzavarini, 2023; Johnston & Leslie, 2019; Martin, 2015; Renoult et al., 2019; Renoult & Rugg, 2020).

The first point of ambiguity in the evolution of semantic memory is the term, *semantic memory*. When Tulving designated semantic memory as a distinct memory system in 1972, *semantics* has already existed as a specialization of linguistics for over a century. Typically, when a linguist refers to *semantics*, they are talking about word meaning. In contrast, when a semantic memory researcher talks about semantics, they are typically referencing *concepts*. This fractionation between linguistic semantics and semantic memory represented an inflection point where *semantics* meant different things to different people. Moreover, the distinction between conceptual semantic versus lexical-semantic knowledge is not trivial (Bierwisch & Schreuder, 1992). Words are not transparently mapped to concepts (Malt, 2020; Malt et al., 2015), and the relationship between language and conceptual knowledge (i.e., linguistic relativity) remains among the most dynamic and contested areas of cognitive science (Abdel Rahman & Melinger, 2009; Boroditsky, 2001, 2009; Lupyan, 2012; Lupyan & Mirman, 2013; Regier & Kay, 2009).

Since its inception, semantic memory research has involved a multidisciplinary pursuit where many of the contributing disciplines have retained their own autonomous methods and scientific vernacular (for models of interdisciplinarity, see also Nicolescu, 2006; Piaget, 1972; Scholz & Steiner, 2015). No “Rosetta Stone” or uniform nomenclature currently exists for translating the meanings of constructs across researchers and disciplines (for discussion and commentaries, see recent work by Calzavarini, 2023). Popper (2005) argued that formal operational definitions of latent constructs (e.g., *mass*) are a necessity for falsification and incremental theory building (see also Bridgman, 1927). Standardization of a scientific lexicon nominally offers a fixed reference for calibrating different perspectives across people and time.

## Neuroscience as a driver of the lexicon of semantic memory

The early post-Tulving period of semantic memory research was shaped by new constraints on biological plausibility and interdisciplinarity (Abrahamsen & Bechtel, 2012; Saffran, 1982). Many of the field’s most vocal and enduring theoretical debates have involved reconciling data from neuroscience, first from patient-based dissociations and more recently from functional neuroimaging (e.g., fMRI, MEG) and neurostimulation paradigms (e.g., TMS, tDCS; Anderson et al., 2019; Binder et al., 2009; Borghesani & Piazza, 2017; Fernandino et al., 2016, 2022; Hauk & Tschentscher, 2013; Huth et al., 2012, 2016; Jefferies, 2013; Kiefer & Pulvermüller, 2012; Kuhnke et al., 2023; Lambon Ralph et al., 2016; Meteyard et al., 2012; Popham et al., 2021; Tang et al., 2023).

One of the most formative discoveries for the emerging field of semantic memory involved Elizabeth Warrington’s (1975) case series of patients who showed a selective impairment of semantic memory (see also Warrington & Shallice, 1984). Snowden and colleagues (1989) later codified the syndrome of *semantic dementia* as emerging from circumscribed, progressive atrophy of the anterior temporal lobes (see also Bozeat et al., 2003; Hodges & Patterson, 2007; Jefferies et al., 2006; Lambon Ralph et al., 2001; Patterson et al., 1994, 2006; Rogers et al., 2004a, 2004b; Rogers et al., 2007; Snowden et al., 1989; Neary et al., 1998; Woollams et al., 2008).^2^ Unlike classical linguistic and/or perceptual access disorders such as aphasia or visual agnosia, semantic dementia is characterized by a relatively homogeneous pattern of impairment across different conceptual domains and modalities (i.e., language comprehension≫ language expression≫ visual object recognition≫ tool use; Bozeat et al., 2003; Hodges et al., 2000; Lambon Ralph et al., 1997; Pulvermüller et al., 2009; Reilly & Peelle, 2008; Snowden et al., 2019; Warrington, 1975). Many have interpreted this pattern of homogeneous impairment as evidence for a conceptual store that subserves all semantically mediated processes (for discussion, see also Borghesani et al., 2022).

Debate regarding the format of conceptual knowledge has persisted for the past half century. Phenomena such as category-specific semantic deficits have added complexity to these deliberations, spawning further arguments about modularity (e.g., Do the distributed subdomains of knowledge fractionate?) and plurality (e.g., Are there multiple semantic systems?; Berthier, 1999; Borgo & Shallice, 2003; Capitani et al., 2003; Caramazza & Mahon, 2006; Damasio et al., 2004; De Renzi & Lucchelli, 1994; Dell et al., 1997; Farah & McClelland, 1991; Gonnerman et al., 1997; Green, 1998; Grossman et al., 2013; G. W. Humphreys & Forde, 2005; G. W. Humphreys & Riddoch, 2006; Jefferies et al., 2004; Kroll et al., 2010; Lambon Ralph et al., 2003, 2007; Mahon et al., 2009; Moss et al., 1998; Price et al., 2003; Sacchett & Humphreys, 1992; Thompson-Schill, 1999; Trumpp et al., 2013; Warrington & Shallice, 1984; Vigliocco et al., 2004). As new empirical questions, new sources of data, and new methodologies have emerged, the lexicon for describing semantic phenomena has expanded in kind.

## Mechanisms for reducing implicit bias

We assembled a workgroup composed of scholars with expertise in semantic memory spanning a variety of disciplines (e.g., psychology, neurology, philosophy, linguistics, speech–language pathology), geographic regions, career stages, and specialties (e.g., neuroimaging, neuropsychology, natural language processing, computational neuroscience). Together we isolated a set of target constructs and crafted succinct definitions via an iterative consensus procedure involving voting, recalibration, and principled individual expressions of dissent.

The process of defining abstract constructs is a uniquely human endeavor. Although standardization offers numerous benefits, there also exists the potential for harm when self-selecting groups of experts impose guidelines on a broader community of stakeholders (for a discussion of the American Psychaitric Association’s efforts to standardize psychiatric diagnoses, see Drescher, 2015; Frances, 2012). It is, therefore, critical to first contextualize the purpose and value of a semantic glossary. This resource is not intended to be prescriptive, but rather to provide a point of reference other researchers might find useful in specifying their own semantic constructs in facilitating cross-disciplinary communication. These definitions do not represent an immutable set of standards, but instead offer benchmarks for criticism and calibration as standards evolve.

In addition to prescriptiveness, another consideration for developing consensus criteria is representativeness. The scientific community investigating semantic phenomena is vast. Any synthesis must include scholars with diverse expertise and opposing perspectives. It is an open question as to who and how many experts should be included in a consensus workgroup. Although the Delphi consensus method outlines considerations for assembling representative workgroups (Linstone & Turoff, 1975), its reliance on anonymity and skilled facilitators is not entirely feasible when expert panelists are readily identifiable by their own unique perspectives. Instead, we opted for a mechanism involving personal interaction, resolution, and whenever possible, compromise among co-authors.

Intersectional bias (implicit and explicit) is another threat both in curating expert panels and in group dynamics within such panels. Workgroup members here were tasked with meeting in small groups by video conference to cooperatively generate consensus definitions. Perceived power imbalances represent another source of bias across numerous demographics (e.g., sex, career stage, nationality, language proficiency, scientific discipline). For example, a female, early career stage, L2 English speaker might be reluctant to disagree with an emeritus distinguished professor. We implemented a formal dissent mechanism to give voice to all members of the workgroup who held principled objections to any definition. Each written dissent was appended to the corresponding construct’s background section.

## Methods

### Workgroup composition and inclusion criteria

Our aim was to assemble a workgroup composed of experts in the study of semantic memory with the following a priori constraints: (1) The panel should be balanced as closely as possible for sex. (2) The panel should include approximately 50 contributors. (3) The panel should reflect a wide range of career experience. (4) The panel should reflect geographic variability of the institutional affiliations of contributors. (5) The panel should represent a variety of theoretical and applied disciplines (e.g., psychology, linguistics, neurology).

Author J.R. initiated recruitment by identifying an initial slate of 30 potential contributors and a preliminary set of 20 target constructs. As the workgroup grew, new panelists offered recommendations for other contributors. In total, we invited 77 scholars to participate (38 female, 39 male; 34 from Europe, 33 from North America, three from South America, three from Oceania, three from Asia, one from Africa). Five authors (two female, three male) began the project but later withdrew, while 20 authors (11 female, nine male) declined or did not respond to the invitation.

The final workgroup was composed of 52 scholars with academic appointments spanning the following fields: cognitive psychology, developmental psychology, linguistics, cognitive neuroscience, neurology, speech–language pathology, neuropsychology, and philosophy. Workgroup members had a range of career experience (i.e., postdoctoral fellow to emeritus professor). Primary academic affiliations spanned 13 countries (i.e., Australia, Belgium, Brazil, Canada, China, France, Germany, Ireland, Italy, Singapore, Switzerland, United Kingdom, United States) and four continents (22 from Europe, 25 from North America, one from South America, two from Oceania, three from Asia). The sex distribution was 26 male and 26 female.

### Procedures for generating definitions

**Fig. 1:**
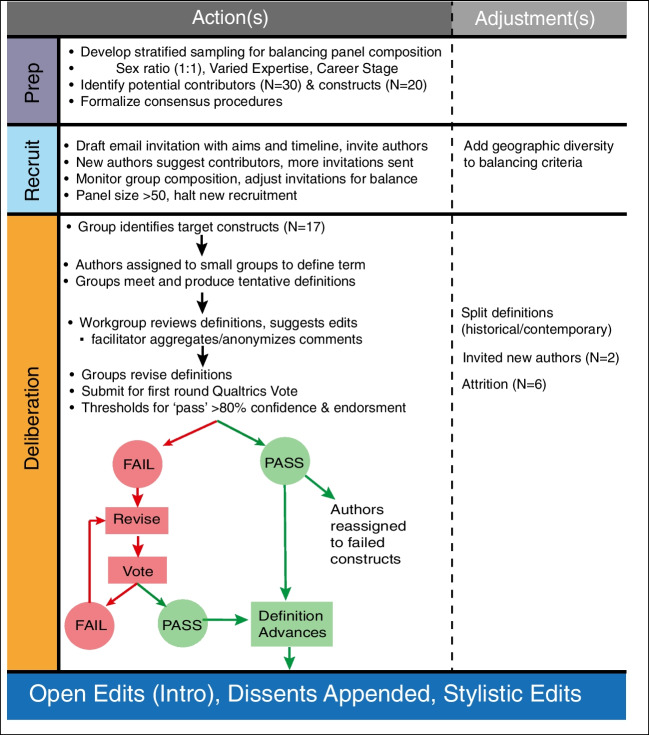
Consensus mechanism flowchart

Figure 1 illustrates a flowchart of the consensus procedures we used to define entries and elicit dissents.

The newly constituted workgroup first settled on a set of target constructs using the base list (*N* = 20) as a starting point. The outcome of this process involved merging all morphological derivatives of modality (e.g., amodal, modality-specific, heteromodal) under a single construct (i.e., modality) and eliminating other constructs (e.g., modularity) as beyond the scope of the project. We ultimately settled on 17 target constructs to be characterized via an iterative procedure (see Fig. 1).

Each author was initially assigned to one group that was tasked with defining one construct. Whenever possible, these assignments were optimized to the content specialization(s) of individual researchers. For example, the group tasked with defining *abstraction* was composed of researchers from several different disciplines who specialize in abstraction and semantic category induction. Groups were instructed to meet via videoconference to: (1) attempt to come to a consensus on a definition for their assigned construct; (2) draft a succinct, unreferenced preliminary definition; (3) produce a longer referenced background section to justify their definition.

After each group completed its first pass, all definitions were distributed to the entire workgroup for review (prior to a formal vote). This was the primary mechanism for integrating wider group feedback into the specialist-generated definitions. Workgroup members were given 1 month to review the first-round suggestions (aggregated and anonymized by the facilitator) and make recommended content revisions.^3^

After all the first-round edits were completed, groups submitted their definitions for a formal vote. The primary mechanism for evaluating agreement and confidence in each of the definitions was a vote administered by Qualtrics. All authors evaluated each construct and indicated endorsement (“I agree with this definition”) and subjective confidence level: “My confidence in adopting this definition is ___ (0 to 100).” Our rationale for assessing both endorsement and confidence is that these metrics yield different but also complementary information (e.g., I might vote for a particular political candidate but not feel great about my decision). We established an a priori target thresholds of > 80% for both endorsement and confidence. Constructs falling below the 80% endorsement and confidence thresholds were redistributed to reconstituted groups for editing and revision. Once these revisions were completed, the constructs were voted on again until cresting the 80% threshold.

### Procedures for reconciling disagreements and expressing dissent

Any author who expressed an irreconcilable difference on any construct was invited to draft a principled dissent regardless of whether that author served on the specialized workgroup that generated the definition. Authors were permitted to dissent as many times as they wished. We appended unedited/uncensored dissents to the background section of the respective construct. Authors were given the option of dissenting anonymously or self-identifying. All dissenters opted to identify. Our rationale for identifying dissenters was as follows:

1. The dissent mechanism links a particular researcher’s perspective with their own body of research, providing insight into their past work while also explaining any reluctance to adopt the consensus definition moving forward.
2. Identified dissents provide a means of recognizing unique perspectives, particularly among early career stage investigators.

### Content of the definitions and supporting material

Each construct included several components: (1) Succinct, unreferenced definition. (2) Endorsement (average) across voters. (3) Confidence rating (average) across voters. (4) Background section explaining the definition (not included in the vote). (6) Dissenting perspectives identified by contributing author.

## Results (Glossary of terms)

The workgroup achieved an average endorsement level of 97.8% (*SD* = 0.02) with an average subjective confidence rating of 84.8% (*SD* = 0.03) after two rounds of voting. Agreement did not differ across sexes (female = 82.38, male = 82.05). A total of 15 dissents were written for eight different constructs by 10 different authors (female = seven, male = three).

## Abstract/Abstractness

**Definition**: (1) (historical): Referring to the quality of a concept (or word meaning) that has no sensory or motor salience (in opposition to concrete) in that it cannot be seen, heard, touched, felt, smelled, tasted or acted upon. (2) (contemporary): The quality of a concept (or word) whose meaning is understood primarily on the basis of language, but also draws from interoceptive experiences, including emotion, introspection, and metacognition. Abstract concepts are often exemplified by perceptually dissimilar associated actions and events.

- **% Endorsement**: 95%; **Confidence (mean)**: 84 (of 100)

**Background:** The traditional definition of abstractness corresponds to the people’s understanding of abstract versus concrete, as revealed in subjective rating studies (Paivio et al., 1968). For most words, there is high agreement among participants about the degree to which the words refer to abstract or concrete concepts. There is also high agreement across rating studies—for instance, agreement between the ratings collected by Brysbaert et al. (2014) and the MRC ratings (Coltheart, 1981), despite differences in instructions given to the participants.

The reason to doubt an abstract versus concrete bipolar dimension in the semantic system is that there is no opposition between language-based and experience-based information. Both sources of information correlate positively with each other and complement each other. For instance, it is possible to produce viable concreteness ratings with embedding-based semantic vectors derived from language corpora (Hollis et al., 2017). Therefore, most information based on experience can also be retrieved based on language use. Some argue that language-based information may be easier to activate, so that the meaning of concrete words is often predominantly based on language information, as it is for abstract words (Gatti et al., 2022; Louwerse, 2018).

Although no formally articulated dichotomous opposition exists, it is widely acknowledged that concrete and abstract concepts both vary along numerous dimensions (Banks & Connell, 2023; Barsalou et al., 2018; Crutch et al., 2013; Reilly et al., 2016; Shallice & Cooper, 2013). Abstract words typically refer to multiple interacting elements rather than a single element. For example, the concept of “cause” includes at least one agent, an action, and at least one patient. It has been argued that abstract words differ in their network organization relative concrete words, with abstract words characterized by higher contextual and thematic/associative salience (Cousins et al., 2018; Crutch & Warrington, 2005) and lower taxonomic cohesion (for recent work on taxonomic relationships among abstract words, see Persichetti et al., 2024). More diffuse organization among abstract words is also associated with lower perceptual similarity among their associative lexical networks (Borghi, 2022; Borghi et al., 2019; Henningsen-Schomers & Pulvermüller, 2022; Langland-Hassan et al., 2021; Lupyan & Mirman, 2013).

Abstract words are typically regarded as *hard words* (Gleitman et al., 2005), and these disproportionate difficulties for abstract words are typically manifested across many domains, including reading, spelling, word recognition, and serial recall (Fini et al., 2021; Sadoski & Paivio, 1994; Sadoski et al., 1997; Villani et al., 2022; Walker & Hulme, 1999). In addition to these objective performance discrepancies, people have reported lower confidence in understanding abstract word meanings and a stronger need for social didactic interactions with other people to acquire abstract word meanings (Fini et al., 2021; Mazzuca et al., 2022; Villani et al., 2019). Words referring to abstract concepts are typically acquired later (Della Rosa et al., 2010; Montefinese et al., 2019; Ponari et al., 2018; Ramey et al., 2013; Reilly & Kean, 2007). In addition, it is thought that abstract words are learned primarily via linguistic input (e.g., definitions, co-occurrence statistics) relative to concrete words that are dually coded both in the language system but also with sensorimotor grounding (Della Rosa et al., 2010; Paivio, 2013; Reggin et al., 2021; Wauters et al., 2003).

Many researchers have underscored the role of language and social interaction in the acquisition, representation, and use of abstract concepts (Borghi, 2023; Dove, 2022). Some authors have also suggested a role for inner speech during abstract word processing (not only overt but also covert language; Borghi & Fernyhough, 2023; Dove, 2019; Fini et al., 2021). Experimental (behavioral and fMRI) and rating studies implicate the involvement of the mouth motor system during abstract word processing, a finding that is consistent with the role of language in abstract meaning (Barca et al., 2017, 2020; Borghi & Zarcone, 2016; Borghi et al., 2011; Dreyer & Pulvermüller, 2018; Ghio et al., 2013; Trumpp et al., 2024).

Abstract meanings are typically associated with more emotional/affective experience (Kousta et al., 2011; Lund et al., 2019; Newcombe et al., 2012; Ponari et al., 2018; Vigliocco et al., 2014), although not all abstract words are affect-laden. Similarly, abstract concepts, particularly emotional ones, are rated as evoking more inner and interoceptive experiences than concrete concepts (Connell et al., 2018; Kelly et al., in press; Lynott et al., 2020; Villani et al., 2021). In addition to language and emotion, abstract concepts are related to visual or motor experience, social constellations, and mental states (Harpaintner et al., 2020; Kiefer et al., 2022; Trumpp et al., 2024; Ulrich et al., 2022).

Different subgroups of abstract concepts have been identified with a differential relevance of specific experiential or linguistic information (Harpaintner et al., 2018; Kiefer & Harpaintner, 2020). Experiments investigating the use of abstract concepts reveal that people prefer starting a conversation with abstract concepts than with concrete concepts (Fini et al., 2023), that they evoke more metaphorical and beat gestures and more words referring to people and introspection (Zdrazilova et al., 2018), and more expressions referring to uncertainty and “why” questions (Villani et al., 2022), consistent with the higher uncertainty they generate.

The meanings of different kinds of abstract concepts might be weighted differently in various dimensions and might have different, even if partially overlapping, neural underpinnings. For example, emotions and interoception might be more crucial for abstract emotional concepts. The kinds of abstract concepts more commonly identified in the literature are the following: Emotions; Numbers + spatiotemporal (magnitude); Social relations; Philosophical-spiritual; Theory of mind/mentalizing; Scientific abstract concepts (Catricalà et al., 2021; Conca, Borsa, et al., 2021; Conca, Catricalà, et al., 2021; Desai et al., 2018; Diveica et al., 2023; Kiefer & Harpaintner, 2020; Kiefer et al., 2022; Mazzuca et al., 2022; Muraki et al., 2020; Muraki et al., 2022a, 2022b; Primativo et al., 2016; Ulrich et al., 2022).

**Dissent #1 for abstract/abstractness (Bolognesi):** The investigation into whether abstract words lack the taxonomic hierarchical organization, a hallmark of many concrete word categories, is currently underway (see Villani et al., 2024). Indeed, certain types of abstract concepts exhibit a greater degree of lexical granularity than others. For instance, within the realm of spiritual concepts, Catholicism can be classified as a type of Christianity, which, in turn, falls under the broader category of monotheistic religions, which is a type of religion, and so forth. Similarly, abstract words and concepts within other social reality domains demonstrate a notably conventionalized taxonomic structure.

**Dissent #2 for abstract/abstractness (Majid):** The contemporary definition of abstractness offered here uses criteria that could apply as well to concepts that typically would be identified as concrete. The proposed criteria are (1) understood based on language; (2) draw from interoception, introspection, and metacognition; and (3) apply to “perceptually dissimilar actions and events.” Arguably all concepts rely on these criteria—for example, even concrete concepts can be perceptually dissimilar (cf. sexual dimorphism in the animal kingdom; e.g., duck, orangutan). For these criteria, it is unclear what would be excluded from the scope. It is also not obvious how to apply “understood *primarily* on the basis of language” across concepts or populations. Are visual concepts concrete for sighted individuals (because they are learned through perception) but abstract for blind people (because they are primarily learned via language input)? Are secondary color concepts (e.g., *sepia, chartreuse*) abstract because we learn about them through language use rather than ostension, but basic color concepts not abstract because they are learned under different conditions? For these reasons, the classic definition of abstract concepts that rests on opposition to the concrete definition is preferable (i.e., abstract concepts are those that are intangible and difficult to perceive directly through the senses).

**Dissent #3 for abstract/abstractness (Bedny):** Not all abstract concepts are learned via language or introspection into affective states. Abstract concepts are present in preverbal infants (cause, object, agent, and “approximately 5”; Carey, 2009; Spelke, 2022). Many abstract concepts have little to do with affect or metacognition (e.g., numbers, logical primitives like “if,” gravity). I would also argue that seemingly sensory concepts are in fact abstract. People who are born blind have rich understanding of color, make generative inferences about color, and use color words appropriately in context (Landau & Gleitman, 1985).

Although I agree that language makes an important contribution to learning abstract ideas, I think this process is more active on the part of the learner than the current definition suggests. The bulk of the evidence on concept acquisition does not point to definitions or tracking co-occurrence statistics as the primary mode of abstract concept acquisition (e.g., Carey, 2009; Gelman, 2009; Keil, 1998; Spelke, 2022). The case of number words is an example that has been well studied. The meanings of number words are acquired in a slow and orderly progression, beginning with one, then two, and so on until the child grasps the successor function. According to several views, the process involves combining information from various prelinguistic conceptual systems (e.g., the approximate number system) and undergoing conceptual change enabled in part via linguistic communication with a numerate community (e.g., Carey, 2009; Feigenson et al., 2004). Inferential conceptual mechanisms, social pragmatic inferences, cultural tools are among the sources of information relevant to the formation and elaboration of abstract concepts.

This definition appears to conflate the role of language in transmitting concepts across minds and representing concepts. Although there is much evidence that language plays an important role in concept transmission, it is much less clear that that abstract concepts are “coded within the language system.” For example, the language system is not the neural substrate of representing number concepts; rather, frontoparietal circuits appear to be involved (e.g., Cantlon et al., 2006; Dehaene, 2011; Piazza, 2010). Nor is it the primary vehicle for representing the minds of other people (e.g., Saxe et al., 2004). What role the language system plays in representing concepts remains to be understood (Fedorenko & Varley, 2016).

## Abstraction

**Definition: **The process of forming general ideas or concepts by extracting similarities and general tendencies from direct experience, language, or other concepts.

- **% Endorsement**: 100%; **Confidence (mean)**: 82 (of 100)

**Background:** The term *abstraction* originated from the Latin word *abstractio*, which is derived from the verb *abstrahĕre*, composed of two Latin elements: *ab*, meaning “away” or “from,” and *trahere*, meaning “to draw” or “to pull.” Therefore, the etymology of *abstraction* reflects the idea of pulling away or separating, emphasizing the cognitive process of distilling essential information or concepts from the complexities of reality.

The term *abstraction* has a rich history with usage that has evolved over time. It can be traced back to ancient Greek philosophy, particularly to the works of Aristotle, who saw the process of abstraction as a way of understanding and categorizing the world. During the Renaissance, Descartes and Locke discussed the role of abstraction in forming general ideas (Laurence & Margolis, 2012; Murdoch et al., 1987). In the early twentieth century, Vygotsky, Piaget, and Bruner studied the development of abstraction throughout childhood, casting abstraction as a fundamental cognitive process. Piaget distinguished between abstraction through associative learning (i.e., pattern and similarity detection) and abstraction through transformation of schema from lower to higher stages of cognitive development (Piaget, 2014). A similar distinction was advanced by French (1995), a computer scientist whose framework of analogy-making describes how different types of conceptual slippages correspond to either (i) abstraction of concrete instances to an abstract schema, (ii) abstraction via transportation of the schema across different situations, or (iii) abstraction that involves transformation of schema to align with a novel context. More recently, Barsalou (2003) identified six distinct types of abstraction, two of which refer to constructs defined elsewhere in this work (i.e., categorical knowledge—see Category/Categorization; abstract concepts—see Abstract/Abstractness), three of which describe the output of the process of abstraction (i.e., summary, schematic, and flexible representations), and finally one that (partially) covers the process we here consider: the ability to generalize across category members.

Abstraction is similar to generalization (Colunga & Smith, 2003), with one difference being that abstraction refers to identifying essential features or properties to form a higher-level representation, whereas generalization refers to the process of transferring knowledge or skills from specific instances or exemplars to new contexts (Son et al., 2008). Abstraction should not be confused with *abstractness*, even though the two variables are positively correlated (Bolognesi & Caselli, 2023; Bolognesi et al., 2020). In fact, abstraction processes can apply to the construction of both concrete and abstract concepts.

Abstraction is often empirically assessed via tasks or measures that require participants (1) to identify common features or properties shared by a group of objects or events, (2) to generalize properties from known to novel items, and (3) to infer and apply abstract rules or schema. Examples include the following:

1. *Categorization tasks,* which typically involve providing a set of instances to the participant who is asked to sort instances into categories or provide the category label of each instance. Such tasks are commonly used in developmental psychology research to investigate children’s categorization abilities (Gopnik & Meltzoff, 1987; Sloutsky & Fisher, 2004), although they are also used to study the nature of expertise by asking experts and novices to categorize physics problems (Chi et al., 1981) and to examine how variability within the category influenced categorizations by manipulating the amount of distortion from prototypical “grid images” that participants were later asked to categorize (Fried & Holyoak, 1984), among many other applications.
2. *Analogical reasoning tasks,* where participants are given an incomplete analogy typically consisting of pairs of conceptual entities (e.g., bread: slice of bread: lemon:?) and have to complete the analogy (i.e., slice of lemon). Note that these conceptual entities do not necessarily need to be linguistic in nature—images of shapes or abstract patterns, or images of people and objects, have been commonly used (e.g., people pieces analogy task; Rattermann & Gentner, 1998; Viskontas et al., 2004). Success in this task relies on the ability to abstract out the common relations that apply to both domains (e.g., the second object is obtained by slicing the first with a knife) and applying the relation to infer the identity of the missing entity.
3. *Novel noun generalization tasks* that involve showing an exemplar and labeling the exemplar (e.g., “This is a /dax/”). The participant is then shown other (novel) objects and asked which objects have the same name (i.e., is also a /dax/). The task measures how individuals generalize a category label to novel instances and is commonly used by developmental psychologists to study the emergence of conceptual categories in child development (Colunga & Smith, 2003; Landau et al., 1988; Soja et al., 1991).
4. *Problem-solving tasks*
5. or tasks that involve *higher order reasoning* about physical systems (Schwartz & Black, 1996), mathematical concepts (Fyfe et al., 2014), or abstract sequences (Kemeny & Lukacs, 2019). The classic study by Schwartz and Black (1996) presented students with problems that led them to solve for the direction of the final gear in a sequence of turning gears and showed how students can transition from a depictive model to inferring the abstract rule that could be used to solve future problems.

**Dissent:** None.

## Action semantics

**Definition:** Action semantics subsumes a collection of diverse neurocognitive representations engaged in meaningful action performance, manipulable object and action recognition, tool use, action categorization, and language about events involving actions.

- **% Endorsement**: 100%; **Confidence (mean)**: 81 (of 100)

**Background:** A diverse array of hierarchically structured neurocognitive representations support action semantics (Grafton & Hamilton, 2007). At lower levels of hierarchy, action semantic representations include embodied/grounded sensory (visuo-somatosensory-kinesthetic) information about how actions should look and feel. These representations subserve action performance and recognition, as well as knowledge of the actions relevant to manipulable objects (e.g., a hammer is used with an oscillating gesture that looks and feels a certain way). For example, the left intraparietal sulcus/supramarginal gyrus (IPL, SMG) and lateral occipital-temporal cortex (LOTC) support action retrieval during recognition of manipulable objects and actions (Garcea & Mahon, 2014; Chao & Martin, 2000; Raffaele et al., 2019). In motor production tasks (e.g., object use or meaningful gesture production), action semantic representations serve as “targets” that guide specific motor plans to achieve the desired sensory states for familiar actions. However, action semantic representations are not motor plans themselves. Rather, these representations include the range of actions that would accomplish the goal of, for example, hammering and the typical actions performed within a given context. Action semantic representations at this embodied level are organized in terms of the similarity of their action features, such that representations with hand and arm trajectories that look and feel similar compete during retrieval (Watson & Buxbaum, 2014). These representations may be implicitly activated when manipulable objects are viewed (Lee et al., 2013), and are distinguishable from actions specified solely by the structural “affordances” of objects: the latter are calculated online and allow appropriate object grasping even when an object is unfamiliar and/or the skilled use associated with it is unknown.

*At higher levels of the hierarchy, action semantics include abstract causal and mentalistic representations of intentions and goals.* Infants perceive actions as intentional and goal-directed within the first few months of life (Liu & Spelke, 2017). Neural systems involved in action processing are sensitive to the unobservable intentional and causal structure of actions (Bi, 2021; Laurence & Margolis, 2012). For example, neural response patterns in the right superior temporal sulcus (rSTS) are sensitive to the distinction between helping and hindering events, reflecting sensitivity to the agent’s social goals (Isik et al., 2017). Regions that respond to language about actions (i.e., action verbs), including the posterior left middle temporal gyrus (pLMTG) represent not only observable physical actions (e.g., running) but also invisible mental ones (e.g., thinking, wanting) and develop invariantly in the face of changes in sensory experience, such as congenital blindness or congenital absence of limbs (Bedny et al., 2008, 2012).

Not all verbs refer to explicit actions (e.g., rusting, existing), and not all actions are strictly verbs (e.g., s*wimming* is my favorite exercise). Verbs are fundamentally grammatical objects defined by their syntactic behavior in sentences, with morphological, argument structure, thematic, and morpho-phonological properties that are partially orthogonal to action semantics (Bird et al., 2000; McRae et al., 1997; Vigliocco et al., 2004). The neural basis of actions and verbs is partially dissociable (Arévalo et al., 2007; Damasio & Tranel, 1993; Hillis et al., 2004; Vigliocco et al., 2004), and the mapping of actions to verbs varies cross-linguistically (e.g., cut-with-scissors and cut-with-knife are distinct, basic-level verbs in Dutch and Mandarin; see Majid et al., 2008).

Action semantic representations at the two main levels of hierarchy interact dynamically during behavior. For example, during a motor action, such as swinging a golf club, the action goals and intentions are translated into the kinematics of the limb movements (Desai et al., 2018; Fernandino et al., 2016). Action semantic representations are not an “all-or-none” phenomenon. That is, not all aspects of our knowledge of “give” or “cut” are retrieved every time an action or manipulable object is viewed or imagined (Lee et al., 2013). Rather, retrieval is influenced by contextual factors, including task goals, social communicative context, current bodily states, affordances, and other cues present in the environment (Xiong et al., 2023).

**Dissent #1 for action semantics (Papeo):** The investigation on the posterior superior temporal sulcus (pSTS) region that seems to selectively respond to, and discriminate between social interaction events (i.e., helping vs. hindering; Isik et al., 2017) is currently ongoing. In effect, discrimination has been reported during visual perception of events (i.e., helping and hindering) that systematically differ for visuospatial properties (e.g., spatial relations between actors, motion trajectories), leaving open the possibility that effects of “social goals” reflect visuoperceptual rather than semantic differences between action events (see Bellot et al., 2021; Pitcher & Ungerleider, 2021). The present observation also highlights a general difficulty in defining the boundary between semantic and perceptual representation, due to both methodological and conceptual limitations of the field (for recent discussion see Hafri & Firestone, 2021).

**Dissent #2 for action semantics (Majid):** There appears to be a categorical error in this definition of action semantics, which includes in it “language about events involving actions.” However, semantics is one component of language that deals with meaning. Other levels of analysis would include, for example, phonology and syntax. So, to define action semantics as including language is a conflation of different linguistic levels. It is an open question—much debated—whether linguistic semantics and nonlinguistic concepts are identical or at least partially distinct.

## Concept

**Definition: **Concepts are coherent, relatively stable (but not static) units of knowledge in long-term memory that provide the elements from which more complex thoughts can be constructed. A concept captures commonalities and distinctions between a set of objects, events, relations, properties, and states. Concepts allow for the transfer and generalization of information without requiring explicit learning of every new instance.^4^

- **% Endorsement**: 98%; **Confidence (mean)**: 90 (of 100)

**Background:** The definition of *concept* in contemporary cognitive neuroscience owes a great deal to Tulving’s (1972) conception of semantic memory as a common substrate for language processing and other cognitive activities. Researchers have offered various characterizations of how concepts serve this functional role. Eleanor Rosch’s (1973) pioneering research on the categorization of everyday objects framed human concepts as those that “provide maximum information with the least cognitive ability.” Clark (1983) defines *concept* as “a set of properties that are associated with each other in memory and thus form a unit.” Murphy (2002) proposes that “concepts are a kind of mental glue, then, in that they tie our past experiences to our present interactions with the world, and because the concepts themselves are connected to our larger knowledge structures.” While Medin and Coley (1998) write, “By concept we mean a mental representation of a category serving multiple functions, one of which is to allow for the determination of whether something belongs to the class. A category refers to the set of entities picked out by the concept.” They distinguish seven categories of functions: categorization, understanding, inference, explanation and reasoning, learning, communication, and combination.

Concepts can be verbal or nonverbal. Nonverbal animals, including human infants, exhibit concepts because they produce untrained responses to novel members of a common class, even when those class members are physically quite distinct (Carey, 2009; Gelman, 1996; Lazareva et al., 2004). For example, 9-month-old infants who discover that a toy wails when tipped will persist in tipping that object when it does not wail and will generalize their tipping action to distinct novel objects that share some properties with the toy but not to dissimilar objects (Baldwin et al., 1993). Preverbal and nonverbal concepts are sometimes called “equivalence classes.” An equivalence class is a subtype of “concept” in which a group of distinct stimuli elicits a common behavioral response (Urcuioli, 2006). Many accounts of concept acquisition propose a continuum from concrete to abstract, or from similarity-based to theory-based, and these distinctions might be useful for characterizing concepts, but they do not neatly map onto stages of evolution, development, or linguistic knowledge (Gelman, 1996).

Concepts are so central that they have been a subject of inquiry since ancient times. The classical theory of concepts, which dates back at least to the ancient Greeks, posited that concepts are definitions built from simpler concepts (e.g., *bachelor* = *unmarried* + *man*). However, a problem for the theory is that precise definitions do not exist for most concepts (e.g., what defines a *game*?; Wittgenstein, 1953). Two influential cognitively oriented theories have avoided this problem by doing away with definitions: Prototype theory holds that concepts are probabilistic: for each concept (e.g., *dog*), a list of features is encoded (e.g., *has four legs, has fur, barks*) and weighted by how frequently it has occurred relative to the target concept in the past (see Rosch & Lloyd, 1978). In contrast, exemplar models not only avoid definitions, but they also suggest that a stored list of features is unnecessary (Medin & Schaffer, 1978; Smith & Medin, 1981). Instead, to decide if something is, for example, a *dog*, we compare it to each of our previous experiences with dogs (stored in mental representations).

Some have questioned whether the term *concept* picks out a productive scientific kind. Miller and Johnson-Laird (1976) write: “Concepts are invisible, impalpable, ill-defined abstractions that have a nasty way of being whatever a theorist needs them to be at the moment” (p. 697). In a more cautious vein, Murphy (2002) notes, “Concepts may have a great variety of forms and contents, and this is part of what has made the field so complex.” In fact, much critique has focused on the overwhelming amount of attention in cognitive science and neuroscience to studying concepts with clear denotations (i.e., objects, events, relations) in contrast to those grounded in social systems (e.g., kinship, marriage, ownership), linguistic systems (e.g., tense, aspect, mood), or logical systems (e.g., conjunction, possibility, necessity). Machery (2009) argued for abandoning the nomenclature of “concept” because the available evidence suggests that there are separate mechanisms associated with exemplars, prototypes, and theories. Less radically, some have suggested that researchers remain justified in using the term but may need to acknowledge that concepts can be complex hybrids (Edwards, 2011; Prinz, 2004).

There have been long-standing debates concerning the flexibility of concepts. Concepts have traditionally been defined in terms of invariant default knowledge that exhibits three characteristic properties: rapid retrieval, automaticity, and context-independence (Machery, 2016). Barsalou (1983) proposed that concepts encompass both context-independent and context-dependent properties. More recently, many researchers have proposed that concepts are flexibly shaped by task and context (Barsalou, 2016; Casasanto & Lupyan, 2015; Connell & Lynott, 2014; Hoenig et al., 2008; Kuhnke et al., 2021; Yee & Thompson-Schill, 2016).

**Dissent #1 for concept (Bedny):** This definition appears to assume that concepts are largely learned from sensory experience. For example, the definition makes a stark distinction between infant’s concepts that are preverbal/nonverbal and those that are verbal. This characterization is not universally agreed upon. There is evidence that some concepts of preverbal infants endure into adulthood and continue to play a role in cognition (e.g., cause, agent, approximate numbers; Carey, 2009; Spelke, 2022). These abstract concepts also serve as building blocks for development and learning through experience, including sensory experience, social learning, and language (Carey, 2009; Gelman, 2009; Gopnik & Wellman, 1992; Keil et al., 1998; Spelke, 2022).

A key feature of concepts that this definition does not sufficiently discuss is their situation within intuitive theories or domains of knowledge (e.g., Carey, 2009; Gopnik et al., 1999; Gopnik & Wellman, 1992; Spelke, 2022). Rather, the definition appears to emphasize feature-based accounts. A large body of evidence suggests that, from early in life, concepts are situated in theory-like causal mental models. Even for young children, not only do dogs have fur and tails, but, unlike chairs and rocks, they also originate from other dogs, eat, breath, and grow. Our concepts of animals fit into an intuitive theory of biology (e.g., Hatano & Inagaki, 1994). Likewise, when reasoning about agents, young infants consider their goals, intentions, and beliefs (e.g., Gopnik & Wellman, 1992; Onishi & Baillargeon, 2005; Woodward, 1998). By contrast, when reasoning about the behavior of inanimate objects, infants rely on an intuitive causal model of physics (Carey, 2009). These mental models also affect how we interpret the perceptual features of objects and have a profound effect on learning (e.g., the motion of an agent might be attributed to goals, whereas that of an object to gravity or the force of another object (Springer & Keil, 1991).

## Concrete/Concreteness

**Definition:** (1) (historical) The extent to which a word or concept evokes an experience grounded within the five Aristotelian basic senses (e.g., vision, audition, olfaction, gustation, tactition; sense as referenced by Locke, 1685). This historical perspective was often used categorically in reference to the distinction between abstract and concrete knowledge. (2) (contemporary) The extent to which a word or concept evokes a (multi)sensory experience encompassing both the classical basic senses but also extending to the chemical senses, interoception, and sense of self (e.g., body awareness and related phenomena).

- **% Endorsement**: 95%; **Confidence (mean)**: 92 (of 100)

**Background:** References to the distinction between abstract and concrete words are pervasive throughout the histories of linguistics and Western philosophy. Modern empirical efforts at measuring and controlling for concreteness effects first involved asking young people to provide subjective ratings of words using Likert scales. These foundational methods were advanced by Alan Paivio (1926–2016) and his many colleagues and collaborators.

The historical definition of concreteness referenced above was derived from the original rating scale reported by Paivio et al. (1968), asking participants to rate the extent to which a word can be experienced through the senses. This operational definition of concreteness served as the gold standard for a vast body of research on concreteness and imageability effects over the subsequent half century (Breedin et al., 1994; Cousins et al., 2018; Hoffman & Lambon Ralph, 2011; Papagno et al., 2009; Plaut & Shallice, 1993; Sadoski & Paivio, 1994; Schwanenflugel & Stowe, 1989). Concreteness ratings are typically derived via Likert-scale ratings reflecting a continuous range of sensory salience rather than a dichotomization of abstract or concrete. For many cognitive scientists today, the meaning of concreteness has evolved to include a wider range of sensory experiences, including sensations initiated within the body (e.g., hunger, emotional pain, interoception). The traditional dichotomy of concreteness as a marker of sensory salience has been replaced with a deeper understanding of abstract words having their own unique representational content (for a critique, see Shallice & Cooper, 2013). One of the challenges involved in manipulating concreteness as an independent variable is the historical drift of this construct and its variable interpretation across different fields (e.g., educational psychology). Since *concreteness* comes with centuries of historical baggage, some researchers have recently moved toward alternative measures of sensorimotor salience (Connell & Lynott, 2012; Muraki et al., 2022a, 2022b; Pexman et al., 2019).

**Dissent #1 for concrete/concreteness (Hoffman):** There are two separate issues at stake in this definition. The first is a measurement issue: What criteria do researchers use to determine how concrete a word is? Unlike many of the constructs defined in this article, concreteness has long been quantified through large-scale rating studies (as has its cousin, imageability). Most language research uses one of these sets of ratings to index concreteness, providing a common operational basis for the construct. Major studies collecting concreteness ratings have used definitions that emphasize the senses through which we experience the external world. For example, Brysbaert et al.’s (2014) ratings for 40,000 English lemmas used the instructions: “*A concrete word … refers to something that exists in reality; you can have immediate experience of it through your senses (smelling, tasting, touching, hearing, seeing) and the actions you do*” (p. 906). Instructions do not typically mention chemical senses, proprioception, or sense of self as determinants of concreteness. Therefore, I would argue that the historical definition is, in practice, what most researchers are using to operationalize concreteness in contemporary research.

The second issue is what types of experience are central to the meanings of the words that people classify as concrete. Here, the contemporary definition acknowledges a growing understanding that experiences of our own internal states (physical, cognitive, and emotional) contribute to semantic representation (Barsalou, 2016; Kiefer & Harpaintner, 2020; Vigliocco et al., 2014). However, it is far from clear that these types of experience are particularly associated with concrete words, as conventionally defined. In fact, many researchers have argued that interoceptive and emotional experiences are more prominent in the representations of abstract words (see Abstractness definition). Ultimately, this debate illustrates the difficulty in reducing the complexity of sensory experience to a single unidimensional construct. Multidimensional approaches may offer a more nuanced way forward (Binder et al., 2016; Connell & Lynott, 2012; Crutch et al., 2013).

**Dissent #2 for concrete/concreteness (Reilly):** Although participants are typically given explicit instructions on how they should rate concreteness, words such as “pain” “spicy,” and “smelly” that index interoceptive or chemosensory states are in fact relatively high in rated concreteness (as are words such as ghost and spirit). One possibility is that words whose meanings are salient in one modality (e.g., hunger) evoke strong contextual associations with concrete words. This phenomenon is evident when people describe odors by anchoring their meaning to source emitters (e.g., “smells like a skunk”). For this reason, I favor the more expansive sense of concreteness as denoting any bodily experienced sensation. Thus, most words are at least somewhat concrete with relatively few exceptions (e.g., the, a, any).

**Dissent #3 for concrete/concreteness (Majid):** A concrete concept has historically been defined as one that is tangible and perceived directly through the senses. While the five-sense model of perception does not accurately reflect our current scientific understanding of the senses, it is important to note that the addition of the “chemical senses” in the contemporary definition has precedent since smell and taste are chemical senses. The background section states that “the meaning of concreteness has evolved to include a wider range of sensory experiences, including sensations initiated within the body (e.g., hunger, emotional pain, interoception),” but these interoceptive states were also used to define abstractness in the earlier definition, making it unclear how these should be used by researchers to identify concrete versus abstract concepts. One remedy would be to maintain the classic definition “perceived directly through the senses” while acknowledging our expanding understanding of the senses to include chemesthesis, proprioception (both of which would have been included under classic “touch”). The sense of self is distinct, however, since as well as including some perceptual elements, it also includes notions of self-awareness, personal identity, consciousness, and so forth, which arguably are not concrete and should not be used to define concreteness.

**Dissent #4 for concrete/concreteness (Bolognesi):** The operationalization of concreteness by means of concreteness ratings has limitations, some of which are described here. A (in my opinion) major limitation that is not mentioned here is the fact that such ratings are typically collected by showing words in isolation, decontextualized from language use. Research has shown that this is problematic especially for polysemous words that have a very concrete and a very abstract sense (Reijnierse et al., 2019), like “side” (concrete surface of an object) and “side” (abstract argumentative standpoint), where both meanings are quite frequent and salient in a speaker’s mind. In fact, “side” has a medium concreteness score with a fairly high standard deviation, suggesting that judgments about the two senses of “side” are conflated in the final concreteness score. Recent studies have started to release datasets of concreteness ratings collected on words shown in context (Montefinese et al., 2023), tackling the different senses outlined above. However, there are other contextual factors that impact the perceived concreteness of a concept, including the actions that are performed with it: is the concreteness of “apple” the same, when we read “I imagined an apple” and “I bit an apple”?

## Embodied cognition versus grounded cognition

**Definition**: (1) (historical) Embodied cognition holds that cognitive functions depend on bodily experiences. In the specific field of semantic cognition, embodied cognition claims that words and concepts are acquired and represented via bodily experiences (i.e., perception and action). (2) (contemporary) Embodied cognition refers to theories claiming that concepts exclusively comprise sensory and motor features represented and processed in modality-specific sensory and motor brain regions. Grounded cognition is the theory that concepts contain perceptual and motor features represented and processed in modality-specific perceptual and motor brain regions. Perceptual features may include internal states such as interoception or emotion, in addition to external sensations. Grounded cognition theories often assume that modality-specific features are complemented by more abstract cross-modal representations.

- **% Endorsement**: 93%; **Confidence (mean)**: 80 (of 100)

**Background:** Embodied and grounded cognition are related terms often used interchangeably. Both embodied and grounded cognition emphasize a crucial role of the human body in conceptual knowledge representation and processing (Barsalou et al., 2008; Pulvermüller, 1999). Embodied and grounded cognition offer a compelling solution to the so called “symbol grounding problem” (faced by amodal theories) that symbols, such as words, can be thought of as empty shells until their meaning is linked to a concrete perceptual or motor referent (Harnad, 1990; Searle, 1980). Grounding (also referred to as symbol grounding or perceptual grounding) specifically refers to symbolic systems such as language where the meanings of words are reified or grounded through bodily experiences (Searle, 1980).

To clearly distinguish the terms *embodied cognition* and *grounded cognition,* we propose to restrict “embodied cognition” to “strong embodiment,” the view that concepts consist *exclusively* of sensory and motor features that are represented and processed in modality-specific sensory and motor brain regions (Gallese & Lakoff, 2005). Note that these modality-specific regions could be higher-level association areas of modality-specific perceptual-motor systems, not necessarily primary sensory-motor cortices (Fernandino et al., 2016; Kiefer et al., 2023).

In contrast, grounded cognition theories are broader and often incorporate internal perceptual modalities, such as introspection, emotion, and mentalizing (Kiefer & Harpaintner, 2020; Vigliocco et al., 2014). Moreover, many grounded cognition theories do not restrict the conceptual system to modality-specific areas but allow for the additional involvement of cross-modal brain regions that integrate modality-specific features into more abstract conceptual representations (Binder & Desai, 2011; Fernandino et al., 2016; Kuhnke et al., 2020, 2023; Simmons and Barsalou, 2003). The latter theories are often also called “hybrid theories” as they incorporate elements from classical embodied cognition theories (i.e., perceptual-motor features represented in modality-specific perceptual-motor areas) and amodal theories (i.e., more abstract, cross-modal features represented in cross-modal convergence zones; Dove, 2023; Kiefer & Pulvermüller, 2012).

**Dissent #1 for embodied cognition versus grounded cognition (Yee):** This dissent is merely about the insertion of the word *exclusively* in the contemporary definition of embodied cognition. In particular, the definition states: “Embodied cognition refers to theories claiming that concepts *exclusively* [emphasis added] comprise sensory and motor features represented and processed in modality-specific sensory and motor brain regions.” Including *exclusively* in this definition turns it into what is often called the “strong” version of embodied cognition (as the background notes). However, I believe that many readers understand the term *embodied cognition* to be a more general one that (by itself) is silent with respect to whether it refers to “strong” or “weak” embodiment (“weak” embodiment allows for the inclusion of components of concepts that are processed elsewhere). More importantly, for those who are new to the field and who may be using the definitions in this article as a guide, I fear that it will create confusion if they attempt to read the existing literature with the view that “embodied cognition” specifically refers to strong embodiment.

I do agree that more clarity is needed regarding what exactly we mean when we use the term *embodied cognition*, as there is certainly a lack of consensus. In fact, in contrast to the definition above, it has been suggested that the “latent majority” view is the weak version (Zwaan, 2014). However, rather than restricting use of the term to cases in which we mean “strong embodiment” (how will we know whether authors are adhering to this?), I suggest that we use explicit language like “a strong version of embodied cognition” or “strong embodiment” when that is what we mean. To give a perhaps clearer example, convincing people to restrict their use of the word “car” to only cases when they mean “red car” would be challenging indeed.

## Event semantics

**Definition:** Event semantics focuses on the perceptual, motor, conceptual, and linguistic representations of events, which, in contrast to objects, typically pertain to how individual entities and the relations between entities persist or change over time. It includes how the continuous flow of experience is segmented into discrete events, with beginnings and endings, along with hierarchical organization.

- **% Endorsement**: 98%; **Confidence (mean)**: 85 (of 100)

**Background:** The linguistics literature on event semantics focuses on how events are represented by words and sentences, and because this literature is both large and heterogeneous, for the present purposes we will list some of the main research topics, since they reflect strong consensus about critical themes. First, a common goal is to determine the most empirically and theoretically coherent way to decompose linguistic representations of events into configurations of semantic features. Some commonly posited basic elements of event structure include AGENT, PATIENT, INSTRUMENT, GOAL, ACT, CAUSE, GO, MANNER, PATH, BE, PLACE, HAVE, BECOME, and STATE. Second, it is widely agreed that there are three broad aspectual types of events: activities, which lack an inherent endpoint (e.g., walk); achievements, which denote the instant at which a state is attained (e.g., win a race); and accomplishments, which extend over time and culminate in a result state (e.g., draw a circle). Third, numerous fine-grained classes and subclasses of event-denoting verbs have been distinguished by a combination of syntactic and semantic criteria. For example, verbs of “breaking” and verbs of “hitting” can both be used in transitive sentences (e.g., The boy broke/hit the window with a rock), but only the former can be used in intransitive sentences with undergoer subjects (e.g., The window broke/*hit). This is because verbs of “breaking” are pure CHANGE OF STATE verbs, whereas verbs of “hitting” encode MOTION followed by CONTACT without entailing a state change. Fourth, related to the previous point, an important aim is to develop semantic explanations of argument structure alternations, which involve different syntactic realizations of similar event structures. Examples include the dative alternation (e.g., Bob gave a ring to Sue/Bob gave Sue a ring), the locative alternation (e.g., Bob loaded hay onto the truck/Bob loaded the truck with hay), and the body-part possessor alternation (e.g., Bob bumped Sue’s arm/Bob bumped Sue on the arm). Fifth, another popular topic concerns the generalized semantic/thematic roles that event participants play. Examples include agent (or actor), patient (or undergoer), experiencer, recipient, and instrument. Sixth, all the topics mentioned above, among many others, are investigated in hundreds of languages around the world, often with the goal of identifying cross-linguistic similarities and differences in the representation of events.

The neuroscientific investigation of event semantics aims to explain how events are represented and mapped in the mind/brain. In the following, we identify the main topics of research concerning different, central aspects of event semantics. First, the study of event semantics in psychology, psycholinguistics, and cognitive and developmental psychology has addressed the universal components of events as a window into the conceptual categories of the human mind. Events are associated with several properties that do not apply to objects. Among them, research has highlighted types of events (e.g., causation, motion, change of state, transfer), temporal properties (e.g., starting moment, ending moment, duration), changes in properties of entities (e.g., size, shape, color, position) or in interactions between entities, and thematic or semantic roles (e.g., agent, patient, goal, instrument), which determine the role of entities in an event and their relation (Rissman & Majid, 2019). How the mind/brain codes event-specific properties, also in relation to sensory, perceptual and motor representations (Kominsky & Scholl, 2020; Papeo, 2020; Strickland & Scholl, 2015), is a focus of current research. Second, the study of event segmentation addresses how the continuous flow of phenomenological experiences is segmented into discrete units, which can be hierarchically structured, with brief, fine-grained events aggregated into extended, coarse-grained events (Kurby & Zacks, 2008; Radvansky & Zacks, 2011). Event segmentation involves shared representations in memory, language, and perception and involves the integration of information on multiple, concurrent timescales. A recent paper (Yates et al., 2023) identifies three main frameworks that have been developed to explain event segmentation: “events as objects,” which emphasizes the similarities between events and (visual) objects; “events as the consequences of prediction error,” which emphasizes the role of prediction in event segmentation; and “events as inferred causal structure,” which focuses on the top-down influence of internal models in event segmentation. Together with the investigation of event boundaries, researchers are now asking questions about the specific contents of events—that is, the parts that are contained within those boundaries (spatiotemporal context, people, goals, states, emotions, etc., and the relationships among them). Third, given that actions are a prominent category of events, the study of event semantics has been informed by the study of behavioral and neural correlates of action and verb processing (Wurm & Caramazza, 2022). Action observation and understanding has been found to consistently implicate a network of occipitotemporal and frontoparietal regions, sometimes called the *action observation network*. While researchers have generally focused on single action events with human agents acting in isolation, more recent work is exploring the networks associated with other types of events like social interactions and natural (i.e., agentless) events. Fourth, research on infants’ cognition investigates the intuitions or expectations that infants have about physical and psychological events, how infants acquire knowledge about events, which aspects of events are privileged in the infant’s mental representation, and how understanding events relates to the sensorimotor experience in the environment (Baillargeon & Wang, 2002; Gergely & Csibra, 2003). Finally, events are fundamental to human experience, as they constitute the stream of experience, the things that are remembered or forgotten in autobiographical memory, and the components of our plans for future action. For this reason, the study of event semantics naturally overlaps with research on perception and sensory-motor processes, episodic and autobiographical memory, and affective neuroscience. Challenges in the study of event semantics primarily reflect the lack of a unified definition of what is an event (i.e., what constitutes an event for an individual and what parts of experience matter). According to recent perspectives (Yates et al., 2023), progress can come from a radical rethinking of what an event is and from recognizing that events are not one thing that can be captured by a single definition, but many things, which may need to be studied separately.

**Dissent #1 for event semantics (Fedorenko):** My primary objection to the consensus definition of *event semantics* concerns the inclusion of *perceptual*, *motor*, and *linguistic* representations, in addition to *conceptual* representations. I use the term to refer selectively to *language-independent and abstract* (not tied to perception and motor control)—that is, conceptual, representations of events.

The reason for separating conceptual representations (for events and more generally) from (1) perception and motor control and (2) linguistic processing is that empirically conceptual representations dissociate from both. First, although we may engage perceptual and motor machinery to process certain kinds of object or event, it is well established that there exist ***conceptual representations that are independent of perceptual and motor processing***. The strongest evidence for the existence of such representation comes from individuals with drastically different perceptual and motor experiences (e.g., individuals who are born blind or without limbs). Despite these experiential differences, these individuals appear to end up with conceptual representations that are remarkably similar to those of individuals with access to the full range of perceptual and motor experiences, as measured using both behavioral approaches (e.g., Bedny et al., 2019; Kim et al., 2019, 2021; Liu et al., 2020) and brain imaging (e.g., Bedny et al., 2012; Striem-Amit et al., 2018; Wang et al., 2020; see Bedny et al., 2008, for complementary fMRI evidence from participants with a full range of perceptual and motor experiences; see Bedny & Caramazza, 2011, for a review). This body of evidence suggests that perceptual and motor systems are not critical to acquiring conceptual knowledge and representing concepts of objects and events.

And second, ***linguistic and conceptual*** (or *semantic*; I use these terms interchangeably) ***processing dissociate*** (again, for events specifically and more generally). At least three sources of evidence support this dissociation. First, prelinguistic infants represent events and make complicated inferences about how agents interact with objects and how objects and agents interact with each other long before they learn words for the constituent event participants and relationships between them (e.g., Hirsh-Pasek & Golinkoff, 2006; Spelke, 2022). Second, some individuals with even severe aphasia (linguistic deficits) lose the ability to interpret and generate linguistic descriptions of objects and events but retain the ability to understand the world (e.g., Antonucci & Reilly, 2008; Chertkow et al., 1997; Saygin et al., 2004; Warren & Dickey, 2021), including making sophisticated judgments about event plausibility, likely event orders, and so on (e.g., Colvin et al., 2019; Dickey & Warren, 2015; Ivanova et al., 2021; Varley & Siegal, 2020). In contrast, conceptual representations can be impaired in other patient populations (e.g., semantic dementia) in the presence of intact linguistic abilities (e.g., Jefferies & Lambon Ralph, 2006; Lambon Ralph et al., 2010). Third, in brain imaging studies, distinct sets of brain areas are activated selectively by linguistic event descriptions versus in an amodal fashion by both linguistic and nonlinguistic (e.g., visual-pictorial) event representations (Baldassano et al., 2018; Ivanova, 2022; Wurm & Caramazza, 2019).

My secondary objection is with the second sentence. A multitude of research questions have been asked and are being asked about how events are represented and processed; it seems peculiar for a general definition to single out a particular research direction (dealing with event segmentation).

**Dissent #2 for event semantics (Majid):** As with “action semantics,” the inclusion of “linguistic representations” to define “semantics” is a conflation of distinct components of language. Event semantics should include within its scope issues of meaning but not, for example, phonology and syntax. So to define action semantics as including language is a conflation of different levels of linguistic analysis. As Fedorenko points out, there are reasons we would want to maintain a distinction between linguistic and nonlinguistic semantics, minimally so we can at least ask as scientists whether these involve identical or distinct representations.

## Lexical semantics

**Definition**: *Lexical semantics* refers to the system of conventionalized meanings of linguistic forms in a language. A *linguistic form* is a sequence of speech sounds (spoken language), manual signs (sign language), visual symbols (orthographic writing systems), or tactile symbols (braille script), or abstractions over these sequences (e.g., sequences of phonemes, graphemes, syllables, morphemes, or words). Lexical meanings can include concepts and relations as well as other shades of meaning conventionally associated with linguistic forms, including affective (e.g., positive or negative sentiment) and social (e.g., class, region, status) information.

- **% Endorsement**: 100%; **Confidence (mean)**: 85 (of 100)

**Background:** Lexical semantics concerns conventionalized form–meaning associations in natural language. The mental system that is thought to represent these form–meaning associations is typically called the *lexicon*. Mappings between linguistic form and meaning in the lexicon are ambiguous and underspecified: a single form can map onto multiple meanings, and a single meaning can map onto multiple forms. For example, a *homonymous* word like *bank* has different unrelated meanings (e.g., side of a river vs. financial institution), whereas a *polysemous* word like *paper* has different related meanings (e.g., piece of paper, newspaper, the building where the publisher sits). The lexicon is structured by systematic relationships that hold between the meanings of lexical entries. These relationships include hypernymy (category), hyponymy (category member), meronymy (part–whole relationship), association, syntagmatic (co-occurrence), and paradigmatic (ability to exchange words in a sentence, often based on synonymy, antonymy, or hypernymy).

Lexical semantics differs from conceptual semantics in its specific focus on *language*, as opposed to other representational modalities (e.g., pictorial) by which meanings can be conveyed and mentally represented. Thus, the meanings of linguistic forms can include not only the concepts to which they refer, but also affective information (e.g., the meanings of *dog* and *cur* differ primarily in affect), social information (e.g., the meanings of *guy* and *gentleman* differ primarily in social register), or related shades of meaning that are specifically associated with linguistic forms (e.g., words) and not inherent to the concepts picked out by those forms. Lexical semantics differs from combinatorial semantics in its specific focus on linguistic meanings that are *stored* in memory, rather than derived or inferred from context.

There are many outstanding questions about the mental lexicon that this consensus definition aims to avoid taking a position on. These questions include the granularity of stored linguistic forms (i.e., whether the lexicon contains morphemes, words, multiword expressions, or some combination of these; e.g., Fiorentino & Poeppel, 2007; Katz & Postal, 1963); the contents of lexical meaning representations (e.g., Cruse, 1986); the format of representations in the lexicon (e.g., Coltheart, 2004); the amount of redundancy in the lexicon (i.e., whether derivable form–meaning relationships can also be stored, e.g., Taft & Forster, 1976); the relationship between the lexicon and the broader semantic system (e.g., Jackendoff, 1992; Sowa, 1993); and the relationship between the lexicon and the grammar (e.g., Bybee, 1998; Goldberg, 1995; Marantz, 1997).

**Dissent:** None.

## Modal//Modality

**Definition:** (1) (historical) From psychophysiology: a specific sensory channel (e.g., color is typically a visual modality). From linguistics: the representational format of any information channel (e.g., newspapers are a print modality). (2) (contemporary) Any discrete channel for transmitting, receiving, and/or representing information including but not limited to primary sensory data. For morphological derivatives of modality (e.g., amodal, heteromodal), we recommend indexing/aligning meanings with common dictionary definitions of these prefixes (e.g., a-, pan-, trans-, hetero-).

- **% Endorsement**: 93%; **Confidence (mean)**: 83 (of 100)

**Background:** Modality is among the most common yet ambiguous terms used in semantic research. For example, many researchers trained in neuroscience and perception link modality with sensory data. That is, modality typically references a discrete sensory channel (e.g., visual modality, auditory modality). In other disciplines such as linguistics, modality is often used in reference to the representational format of a particular stimulus (e.g., newspapers as a print modality). The challenge of converging upon a broad consensus for *modality* is that many subdisciplines of cognitive science have cultivated theories premised on their own unique interpretations of this term (for a recent discussion and alternate proposal for disambiguation, see Raia, 2023). The contemporary definition of modality proposed here represents an amalgamation of perspectives. Namely, we propose that modality references any discrete information channel for either the transmission or representation of information including but not limited to primary sensory data. Thus, vision and orthography could both be considered modalities. Vision is a sensory modality, whereas orthography is a representational modality. Vision and print are both channels dedicated to either receiving or transmitting information. For clarity, we suggest that *sensory modality* be consistently used when limiting to primary sensory data, and *representational modality* used when any dimension (not limited to sensory data) is intended (for a distinction between input modality and representational modality, see Kiefer et al., 2023).

The term *modality* has numerous morphological derivatives. Many of these constructs have featured prominently in a longstanding debate over semantic organization in the human brain. Proponents of embodied theories hold that semantic memory is grounded in modality-specific systems distributed across sensory and motor cortices (Hoffman & Lambon Ralph, 2013; Jefferies et al., 2010; Machery, 2016; Patterson & Lambon Ralph, 2016; Rogers et al., 2004a, 2004b). Another prominent perspective holds that semantic knowledge is mediated by amodal symbols (Hoffman et al., 2018; Machery, 2016; Patterson & Lambon Ralph, 2016; Patterson et al., 2007).

Inflected derivatives of *modality* often index semantic phenomena in opaque ways that diverge from standard dictionary definitions (see Calzavarini, 2023). For example, an unfamiliar researcher might assume that *amodal* means “no modality” since the English morpheme *a-* typically denotes away from, lacking, or without (e.g., asexual, atheist, amoral). However, this is not always the case. Descriptions of commonly used derivatives of modality follow:

- Amodal: Not directly tied to physical aspects of the environment (e.g., not topographically organized).
- Crossmodal: Includes processing from two or more modalities, often referring to perceptual processes occurring within the brain. For example, auditory cortex is typically responsive to both auditory and visual speech information.
- Heteromodal: Synonym for multimodal (see multimodal).
- Modality-invariant: Areas of the brain or of a semantic space that are recruited for a particular target concept regardless of its sensory or representational modality.
- Modality-specific: (Syn: unimodal) Responding to one and only one modality.
- Modality-preferential: Responding more to one modality than others (but may still show a response to more than one modality, in contrast with modality-specific).
- Multimodal: Responding to and integrating across more than one sensory and/or representational modality.
- Polymodal: Synonym for multimodal (see multimodal).
- Supramodal: Synonym for amodal (see amodal).
- Transmodal: Synonym for modality-invariant.

**Dissent #1 for modal/modality (Bolognesi):** From my perspective, ambiguity around the meaning(s) of *modality* is growing, as evident in recent debates in cognitive semiotics and cognitive linguistics (e.g., Bolognesi & Werkmann Horvat, 2022; Stampoulidis, 2020). Printed newspaper articles (the example mentioned in the definition) use primarily written linguistic signs to convey meaning. Print engages visual sensory channels converging upon modality-specific representations in mind. Therefore, the distinction between *sensory* modality and *representational* modality does not resolve ambiguity associated with “modality” because the term *representation* is itself also ambiguous and can refer to both the semiotic system in which a message is expressed and its corresponding conceptual representation.

Rather than representational and sensory modality, a better distinction would be between (1) semiotic systems of expression to define the system of signs through which a message is conveyed—often the research focus of semiotic and linguistic approaches—and (2) sensory modalities to refer to the channels through which messages are processed—often the research focus of cognitive scientific approaches.

**Dissent #2 for modal/modality (Bi):** I oppose defining modal/modality to include both sensorimotor and representational components. While sensory modalities of the brain are clear-cut (for the human brain, sensory: vision, audition, haptic, olfactory, taste; motor), what constitutes a representational “modality” is highly debatable and open-ended. Using modality to also refer to the latter is counterproductive. It would be clearer to follow the neuroscientific convention to use modal/modality for sensory channels, and use other ways to clarify the different types of representational contents (e.g., “representational content” or “information content”). That is, visual modality would mean the visual sensory channel, which can convey information computing various types of content such as shape, color and texture of objects or forms of written language (orthography).

## Semantic control

**Definition**: The set of executive control processes that regulate the activation and deployment of semantic knowledge. These allow flexible, context- and task-appropriate responses by ensuring that only relevant aspects of semantic representations are used to direct thought and behavior.

- **% Endorsement**: 100%; **Confidence (mean)**: 81 (of 100)

**Background:** The contemporary study of semantic control emerged from neuropsychological studies of “semantic access” (Campanella et al., 2009; Warrington & Shallice, 1979) and “refractory access” deficits (Warrington & Cipolotti, 1996; Warrington & Crutch, 2004). This work led to the establishment of a double dissociation between deficits of semantic control in semantic aphasia versus long-term conceptual knowledge representation in semantic dementia (Jefferies & Lambon Ralph, 2006). People with semantic aphasia have difficulty regulating semantic cognition across verbal and nonverbal tasks in different contexts (e.g., resolving lexical ambiguity in the context of distractors). Semantic aphasia is typically associated with left hemisphere cerebrovascular accidents impacting frontoparietal and/or posterior temporal lobe regions (Thompson et al., 2022).

Early neuropsychological studies implicating semantic control later converged with functional neuroimaging studies demonstrating parametric modulation (upregulation) of left inferior frontal gyrus (LIFG) during executively demanding semantic tasks. In a seminal study, Thompson-Schill and colleagues (1997) reported LIFG activity mediated by competition between active semantic representations. The authors proposed that this region mediated top-down selection of relevant semantic knowledge. Badre and Wagner (2002) later argued that LIFG was engaged in effortful retrieval of semantic knowledge as well as competition resolution. These authors coined the term *semantic control* to refer to these processes. It is important to note, however, that LIFG damage is not universally associated with difficulty resolving lexical-semantic competition (Britt et al., 2016). Further, fMRI studies have identified a distributed network of regions that are sensitive to semantic control demands, including LIFG (including a large swathe of pars triangularis, orbitalis, and opercularis) and left posterior temporal cortex (including posterior superior temporal sulcus, middle temporal gyrus, and inferior temporal gyrus; Jackson, 2021; Noonan et al., 2013). Neurostimulation of these regions affects ambiguity resolution and the efficiency with which weak associations can be retrieved (Davey et al., 2015; Whitney et al., 2011). Additionally, neuroimaging studies consistently identify similar effects in bilateral dorsomedial prefrontal cortex (centered on presupplementary motor area) and right IFG (Jackson, 2021), although these areas have received less attention in the literature.

What is semantic control and why do we need it? Representations of a concept consist of a multitude of features and associations which are unlikely to all be applicable to the current situation, and indeed some may directly counteract the current aim. For instance, we normally think of dogs as friendly family pets, but if we encounter one accompanying a security guard, this dominant information will not support appropriate behavior. Semantic control processes act on semantic representations in a top-down fashion to shape activation in the semantic system and produce a conceptual structure that suits the needs of the current context (Zhang et al., 2021). Context can include an individual’s current task or goal but also the wider situation in which processing is taking place (e.g., linguistic or environmental context). Semantic control processes are integral to the normal operation of the semantic system and are assumed to be engaged to some extent in all forms of semantic processing. However, they are most essential when automatic stimulus-driven processing alone does not lead to the context-appropriate aspects of meaning becoming most strongly activated (Wang et al., 2020). There are two sets of circumstances which have been investigated.

First, when automatic stimulus-driven processing fails to activate context-relevant knowledge, controlled retrieval processes are thought to engage a more effortful, internal search for relevant semantic information (Badre & Wagner, 2002; Hoffman, 2018). This may be important, for example, when people need to access less frequent meanings of ambiguous words or to search for novel or less salient associations between concepts. The second case is when multiple semantic representations (i.e., multiple concepts or features) are strongly activated and compete to influence behavior. Here, semantic selection processes are thought to boost the activation of context-appropriate representations and inhibit those that are not currently relevant (Jackson et al., 2021). This may be important, for example, when people make decisions based on specific properties of concepts or simply when tailoring their responses to only include a particular subset of information. Mechanistic accounts of these controlled retrieval (Hoffman et al., 2018) and semantic selection (Jackson et al., 2021) processes can simulate both typical and impaired semantic control. Although these are interconnected processes, there is some evidence that they may have distinct neural and behavioral correlates (Badre et al., 2005). However, the degree to which they are served by distinct neural systems remains an open question.

Current research is focusing on how semantic control processes relate to domain-general executive functions (e.g., Chiou et al., 2023; Gao et al., 2021; e.g., G. F. Humphreys & Lambon Ralph, 2017) and to cognitive control in episodic memory tasks (Vatansever et al., 2021). The left-lateralized semantic control network only partially overlaps the multiple-demand network of regions that respond to increased control demands across a broad set of cognitive domains (Jackson, 2021). Moreover, the key regions demonstrated to be necessary for semantic control—LIFG and posterior middle temporal gyrus—are not part of the core multiple-demand network, suggesting they play a more specialized role in regulating activation of semantic knowledge. Notably, these regions have highly left-lateralized patterns of connectivity (Alam et al., 2019) unlike the more bilateral multiple-demand network. Directly comparing the neural correlates of semantic and domain-general control while separating the effects of difficulty, task and stimulus type will be critical to understand to what extent these processes are neurally and computationally distinct.

Finally, most work on semantic control has used manipulations of verbal stimuli, and much less is known about how the regulation of nonverbal knowledge is achieved. People with aphasia who have concurrent semantic control deficits have also been reported to experience parallel deficits in regulating object use, suggesting shared control processes for verbal and nonverbal knowledge (Corbett et al., 2009). However, the relatively large lesions in such cases could mean that patients had sustained damage to neighboring but distinct systems. Few fMRI studies have investigated semantic control demands in non-verbal semantic tasks (but see Krieger-Redwood et al., 2015) and this is a key target for future research. In addition, within the verbal domain, regions implicated in semantic control do not appear to be engaged in the control of executively demanding phonological processing (Hodgson & Lambon Ralph, 2021; Snyder et al., 2007). This suggests that semantic control cannot simply be equated to control over all verbal stimuli.

Experimental manipulations of semantic control typically involve some combination of reducing the accessibility of task-relevant semantic knowledge while increasing the salience of irrelevant knowledge. For example, accessing less frequent meanings of ambiguous words is thought to place high demands on semantic control, both because the required knowledge is unlikely to be activated automatically during word processing and because strong activation of more dominant meanings must be inhibited. Tasks with similar demands include presenting multiple comparison stimuli (typically words) to probe knowledge for weak semantic associations and feature selection tasks where participants match items based on specific properties (e.g., color) while ignoring irrelevant semantic associations.

**Dissent:** None.

## Semantic dimension

**Definition**: Any variable used for differentiating exemplars (e.g., axe vs. spoon) across any given aspect of meaning (e.g., capacity for inflicting harm). Semantic dimensions are often but not always continuous (e.g., pleasantness vs. animacy). In high dimensional semantic space models, knowledge of the constituent semantic dimensions is essential for determining the coordinate location of any exemplar and computing its distance to all other exemplars.

- **% Endorsement**: 95%; **Confidence (mean)**: 87 (of 100)

**Background:** Throughout the early 1970s to the present, cognitive scientists focused on semantic features in defining category boundaries and constraining word and object knowledge (Breedin et al., 1998; Caramazza & Shelton, 1998; Cree et al., 2006; Garrard et al., 2005; McRae et al., 2005; Rogers et al., 2004a, 2004b). Semantic features typically reflect the binary presence or absence of a particular attribute (e.g., has fur, has a tail). The past decade has seen a new class of models premised upon characterizing concepts using many continuous dimensions such as color salience, arousal, or valence.

The dimensions that comprise experiential semantic models are typically derived through subjective ratings. For example, the Lancaster Sensorimotor Norms reflect salience of dimensions such as color, olfaction, interoception, and hand/arm associations for tens of thousands of words as rated by many human participants (Lynott et al., 2020) and across many languages (I.-H. Chen et al., 2019; Speed & Brysbaert, 2022). Each of these continuous variables constitutes a single dimension. These individual factors are typically combined to form high dimensional semantic spaces (Banks & Connell, 2023; Binder et al., 2016; Crutch et al., 2013; Reilly et al., 2016, 2023).

Word embeddings represent another high dimensional approach to specifying word meaning. However, the elements within vectors that comprise embedding models represent abstract mathematical constructs rather than meaningful psychological variables. For example, many embedding models characterize the meanings of words along 300 dimensions, none of which in isolation is psychologically analogous to semantic dimensions such as color or disgust that characterize more experiential semantic models (for discussion, see Reilly et al., 2023).

**Dissent #1 for semantic dimension (Lupyan and Fedorenko):** Our primary objection with the current definition is the focus on the interpretability of the dimensions. There are two reasons for this objection. First, the interpretability of semantic dimensions does not seem to be a prerequisite for their success in capturing human semantic judgments. In particular, self-supervised word embedding models, like word2vec (Mikolov et al., 2013) and GloVe (Pennington et al., 2014) are highly successful at capturing human behavioral data on semantic tasks (e.g., Pereira et al., 2016). At the core of these models is the idea that words that occur in similar linguistic contexts have similar meanings (Firth, 1957; Landauer, 1997). Some basic versions of word-embedding models can have interpretable dimensions (e.g., how often a given object concept co-occurs with a particular action verb like “eat”; Mitchell et al., 2008). The most general and successful versions of these models, however, involve computing 200–300 latent dimensions, which are no longer readily interpretable, although it seems possible to project them onto more interpretable dimensions, such as concreteness, valence, and arousal.

And second, interpretable semantic dimensions are generally limited: a) they tend to only characterize a subset of concepts (e.g., you one rate object concepts for animacy, but not a relational concept like “below”); b) they are often generated by researchers in a somewhat ad hoc way (e.g., Binder et al., 2016) and, as a result, have baked into them potentially incorrect theoretical assumptions about the nature and organization of concepts; and c) they require large amounts of human behavioral data, which makes them more difficult to generalize to new populations (e.g., individuals living different cultures; Blasi et al., 2022). The fact that these researcher-generated dimensions are easy to understand in no way implies that they reflect the true dimensional structure of people’s semantic space.

As a result, we question the requirement of semantic dimensions to be a priori known or readily understandable. In fact, the dimensional structure obtained from more opaque methods like self-supervised word embedding models may turn out to be a better characterization of the human semantic space, and the phenomenal success of modern-day language models (Radford et al., 2019), which at their core rely on patterns of word co-occurrences, indeed suggests that this is likely to be the case.

## Semantic distance/Semantic similarity

**Definition**: A quantitative measure of similarity/distance between two words (or concepts) situated within an *n*-dimensional semantic space.

- **% Endorsement**: 98%; **Confidence (mean)**: 85 (of 100)

**Background:** Semantic distance/similarity provides an empirical measure of semantic relatedness between two words. Within high dimensional semantic models (e.g., experiential models, embedding models), semantic distance (or similarity) is typically reported as the cosine of the angle between the corresponding semantic vectors for two words (Landauer & Dumais, 1997; Pennington et al., 2014). The angle between two identical vectors is zero degrees with a corresponding cosine value of 1. As such, a cosine value of 1 indicates zero pairwise semantic distance as would be encountered when contrasting one word against itself. Cosine values are bounded between 1 (no distance) and − 1 (maximal distance; anticorrelated). Cosine values near zero indicate high semantic distance (i.e., unrelatedeness) between word pairs.

Semantic distance is only interpretable relative to the unique dimensions of a given semantic space. Consider, for example, two hypothetical semantic spaces such as (1) potential kitchen implements and (2) potential weapons. Many of us would judge knives and guns as semantically distant in “potential kitchen implement” space and as semantically similar in “potential weapon” space. Thus, semantic distance is a relative metric inextricably tied to a semantic space. Describing two entities as “semantically distant” or “semantically related” leads to underspecification unless the corresponding semantic space is also referenced (e.g., knives and guns are semantically distant in their utility as kitchen implements).

**Dissent:** None.

## Semantic feature

**Definition: **A component or element that relates to a concept or expresses a relation with other concepts. A concept can therefore be approximated as a collection of such features. Semantic features capture a wide range of information characteristics of a concept covering taxonomic relations, perceptual properties, function, behavior, thematic roles, and introspective features. Features are typically binary (present or absent for a concept) but can be weighted by criteria such as salience (e.g., [has wings] is important for BIRD) or context dependency (e.g., BIRD sometimes [is pretty]). Certain features also tend to co-occur among category members (e.g., [has wings], [has beak], [can fly]).

- **% Endorsement**: 98%; **Confidence (mean)**: 89 (of 100)

**Background:** Semantic features have a long tradition in both philosophy, psychology, and computer science. Classical views (e.g., Aristotle) considered concepts as being defined by necessary and sufficient features, so that any given concept could be completely defined by providing the full list of its constituent features. In this way, semantic features can allow concepts to be structured into categories according to how their featural representations overlap. This idea was developed in work that viewed the human conceptual system in terms of taxonomic hierarchies (Collins & Quillian, 1969), and was further extended by more modern theories that built extensive concept-feature datasets, where semantic similarity between concepts could be derived by examining the extent of shared features between pairs of concepts (Malt & Smith, 1984). This led to further efforts to collate large-scale sets of semantic feature norms (Buchanan et al., 2019; Harpaintner et al., 2020; McRae et al., 2005), where participants would generate as many features as they could for individual concepts, providing list or vector-like representations for concepts and their features.

Semantic features can be obtained relatively easily from non-specialists, with simple instructions to generate common properties of each concept in a list. In some cases, semantic features are obtained from experts, such as linguists, to build knowledge graphs or semantic networks such as WordNet (Miller, 1995). Once a set of concept-feature lists is collated, similarity between concepts can be calculated by methods such as the cosine of the angle between feature-frequency vectors. For example, two concepts with completely overlapping features would have a cosine similarity value of 1, while two concepts with no overlapping features would have a cosine similarity of 0. There is some debate regarding whether such featural similarity is the best way of estimating semantic similarity or whether alternative, nonfeatural methods are more effective (see also semantic space definition; Wingfield & Connell, 2022). Nonetheless, the featural similarity approach makes semantic features useful when trying to investigate behavior related to phenomena in language processing (comprehension and production) and conceptual representation. Evidence for the utility of semantic features comes from a broad range of studies, from modelling semantic priming (Cree et al., 1999) and category-specific deficits (Tyler et al., 2000; Vinson et al., 2003; Warrington & Shallice, 1984), to investigating the source of false recognition memory (Montefinese et al., 2015).

Semantic features have known limitations. Instructing participants to produce common properties for a concept prioritizes features that are more easily verbalized. As a result, feature lists are affected by the lexical specificity of a language and individual vocabularies of participants and might underestimate conceptual diversity among a group of speakers. Features are also easily generated for concrete nouns but less straightforward to verbalize for abstract nouns and other parts of speech such as verbs and adjectives. Collecting and norming feature lists is also labor intensive, meaning that coverage remains limited. For instance, the largest set of norms to date reported by Buchanan and colleagues (2019) compiles features for nearly 4,500 concepts, which—while extremely useful—is still well short of an adult-level vocabulary of approximately 40,000 words (Brysbaert et al., 2016). Consequently, it is unclear whether semantic features can in isolation provide a comprehensive picture of semantic memory (for critique of feature-based approaches, see Jackendoff, 1987).

**Dissent:** None.

## Semantic representation

**Definition: **The cognitive and neural manifestation of the information content of semantic knowledge, which is the structured knowledge stored in long-term memory (i.e., semantic memory).

- **% Endorsement**: 98%; **Confidence (mean)**: 84 (of 100).

**Background:** From the moment we are born and over the course of our lifetimes, we accumulate massive amounts of knowledge that encompasses knowledge of specific objects and entities (e.g., a cat or a chair), situations (e.g., a birthday party), abstract ideas (e.g., freedom), emotions (e.g., happiness), understanding of general facts (e.g., why people pay taxes), or social norms (e.g., what to wear to a wedding), as well as parts of our knowledge of the world that do not easily map onto a label or a verbal description (e.g., a particular spatial layout). ***Semantic representation*** refers to the currently active subset of this knowledge (the cognitive manifestation or thought about a specific component of semantic memory). The term can vary in its scope: it can be discrete or graded and it can refer broadly to an overarching subset of semantic knowledge about an aspect of the world, or more narrowly to a particular context-relevant feature of an object or event.

Semantic representations (1) are short-lived (time-limited), (2) can get activated by diverse perceptual inputs (a picture of a cat, the sound of a “meow,” the smell of a litter box, etc.), linguistic inputs (the word *cat*), or internal thought processes (a memory of a childhood pet), and (3) are often tailored to the demands of the current situation. For example, to decide whether a cat is smaller than a microwave when playing Twenty Questions, one needs to activate one’s semantic representations of a cat and a microwave, focusing on their sizes. Similarly, to decide whether to adopt a new cat, one needs to activate one’s semantic representation of a cat, but in this case, one may instead focus on the cuteness and cuddliness of cats or the fact that they shed and can scratch furniture. Thus, certain aspects of a semantic representation (i.e., perceptual, functional, situational, etc.) may be more or less salient in particular contexts (Hoenig et al., 2008; Kiefer & Pulvermüller, 2012). In this way, semantic representations can provide a type of interface that binds perception, action, language, and general knowledge. Sometimes, however, semantic representation can refer to context-independent thoughts that pertain to a subset of our world knowledge (e.g., our general knowledge about cats).

Some, but not all, semantic representations are associated with verbal labels. In this case, individuals must have a mapping between labels and different subsets of semantic knowledge. Importantly, however, verbal labels are not part of the semantic representations. Instead, they constitute a separate, language-specific system that may function in parallel with the system that stores our world knowledge but is independent of it. For example, some animals, preverbal infants, individuals with no access to language (e.g., deaf individuals growing up without access to sign language), or individuals with aphasia who have lost access to labels can have semantic representations even though they do not have labels for them. (Note that some linguists and psycholinguists have used the term *semantic representation* to refer to representations of specifically linguistic meaning; we believe this usage can lead to confusion, and we therefore advocate abandoning this usage of the term.)

We have here focused on the cognitive science perspective. Of course, particularly within cognitive neuroscience and cognitive neuropsychology approaches, semantic representations must also manifest as patterns of neural activity, but the 1:1 correspondence between the cognitive and neural manifestations of semantic representations remains debated.

**Dissent #1 for semantic representation (Majid):** The definition provided specifies semantic representations are the “cognitive and neural manifestation” of knowledge, which by definition rules out the possibility that artificial intelligence could have semantic representations. Rather than stipulating this to be the case, I believe this should be a matter of scientific inquiry. In defense of maintaining a distinction between linguistic “semantic representation” and nonlinguistic “conceptual representation,” there are enough documented cases where these dissociate that it is relevant and helpful to maintain the separation.

**Dissent #2 for semantic representation (Bedny):** The current definition describes representations as ephemeral “short-lived” states. This is one use of this term. Representation can also refer to the knowledge stored in long-term memory. The representation of the concept “dog” is the long-term memory that enables us to think about dogs across contexts. When we say that a representation is acquired or present from birth, we are referring to this long-term memory, not to a short-lived state.

This definition describes semantic representations as fully learned and sensory focused. But this need not be the case. There is evidence that some representations (e.g., approximate number representations, representations of agents, objects) are present from birth (Spelke, 2022). Experience then serves to augment an existing representational skeleton. Regardless of one’s position in this theoretical space, we should not define semantic representations to exclude innate representations.

## Semantic space

**Definition**: A latent topography bounded by different aspects of meaning (e.g., valence, arousal, animacy). Semantic spaces provide a coordinate system for situating target concepts and deriving their distances to other words or concepts. Embedding-derived semantic spaces typically distribute a target concept across numerous hyperparameters, whereas the dimensions that comprise experiential semantic spaces are psychologically meaningful in isolation (e.g., color, fear). Semantic spaces are often but not always neurobiologically plausible.

- **% Endorsement**: 97%; **Confidence (mean)**: 84 (of 100)

**Background:** Concepts are composed of many pieces of information, including features (e.g., “has a tail”) and dimensions (e.g., pleasantness). Semantic spaces provide a framework for decomposing words into vectors that capture meaning. The length of these vectors varies as a function of the dimensionality of the semantic space used to define them. For example, a two-dimensional semantic space constrained by valence and arousal would yield a vector of length two applied to any number of words. Although such low dimensional semantic spaces are indeed possible, their utility is limited with respect to explaining real-world semantic phenomena (e.g., category-specific semantic impairments).

**Fig. 2:**
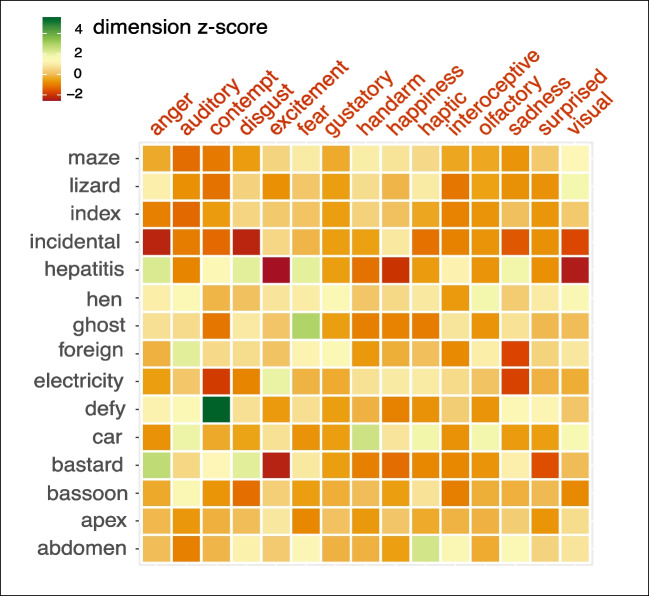
A 15-dimension Experiential Semantic Space (Semdist15). *Note*. Semdist15 is a freely available database via the Open Science Foundation or as part of the semdistflow R package. Download at https://osf.io/5bntg/

Figure 2 illustrates a 15-dimension semantic space that characterizes more than 80,000 English words across key sensorimotor and affective dimensions (Reilly et al., 2023). Each cell in this matrix represents the salience of each word on each dimension (*z* scored). Each row of this matrix represents a discrete semantic vector reflecting the salience of a given word (e.g., *maze*, *lizard*, *index*) across the specified dimensions. Semantic distance between any two words (e.g., maze:lizard) can be derived by computing the cosine distance between their respective vectors.

Semantic spaces could be composed of a potentially infinite number of dimensions. An optimal semantic space would approximate the latent structure of semantic memory. Many efforts at specifying the dimensionality of semantic spaces involve educated guesses about which dimensions should be included that best account for semantic phenomena such as neuropsychological dissociations (Crutch et al., 2013) and decoding the meanings of words and utterances from multivariate brain imaging signals (Fernandino et al., 2022; Huth et al., 2012, 2016; Wang et al., 2020).

Two broad classes of semantic space models have recently evolved. Experiential semantic models are characterized by psychologically meaningful dimensions (e.g., color, emotion) typically reflecting human subjective ratings (e.g., rate the extent to which this word makes you think of color; Banks & Connell, 2023; Binder et al., 2016; Troche et al., 2017). In contrast, word embedding models yield high dimensional semantic spaces characterized by hundreds of hyperparameters generated from co-occurrence statistics in large natural language corpora. A word such as *maze* depicted in Fig. 1 is represented by a 15-element vector in an experiential semantic model (SemDist15), whereas the semantic vector for *maze* generated by GLoVe (Pennington et al., 2014) would span 300 parameters. In experiential models, a researcher manually selects the dimensions, whereas the elements that comprise embedding models are abstract mathematical constructs agnostic to human intuition. The semantic distances generated by experiential and embedding models are strongly correlated, but it has been argued that they index different information about taxonomic and thematic semantic relationships (but see Grand et al., 2022; Reilly et al., 2023).

**Dissent:** None.

## Simulation

**Definition**: Simulation is the prerunning or rerunning of a process outside of the proximate context that normally compels or cues that process to run. Simulation can include input (perceptual), output (motor), and interoceptive (affective and cognitive) processes and can be explicit and intentional or implicit and automatic. An example of explicit and intentional simulation is motor imagery or perceptual imagery. An example of implicit and automatic simulation would be perceptual activity during comprehension of sentences describing sensory events, or motor activity during observations of others’ actions (hand actions, speech).

- **% Endorsement**: 100%; **Confidence (mean)**: 85 (of 100)

**Background:** Simulation has played a prominent role in research within the embodiment framework—the idea that the format of abstract content is sensorimotor. Simulation is the mechanism that makes it possible for cognitive content and conceptual representations to plausibly be distributed over perceptual, motor, and cross-modal association systems. On such strong embodied views, conceptual content is the rerunning of sensory, motor, and interoceptive processes that are engaged during perception, action, and interoceptive experience.

Simulation has also been closely aligned with motor theories of perception, originally formulated in the speech domain (Galantucci et al., 2006; Liberman et al., 1967) and extended to manual actions by research on the mirror neuron system (Rizzolatti & Craighero, 2004). In both contexts, the idea is that motor processes corresponding to the perceived action are run automatically (and implicitly), and that the simulation of those motor processes constitutes (causally) part of the series of processes that constitute “recognition” or “understanding.”

Simulation is also used in a more abstract manner—where what is being simulated are abstract, more cognitively elaborate, and temporally extended representations, such as proprioceptive representations of the self (e.g., body schema), physical events (e.g., melting), and symbolic mathematical operations (Borghi & Cimatti, 2010; Lakoff & Núñez, 2000; Rueschemeyer et al., 2010).

There is little debate that simulation is one of the ways the brain builds predictions about the body and the world, that it is a critical aspect of mental imagery (Decety, 1996; Moulton & Kosslyn, 2009) and that it can play a role in learning and memory (Liu et al., 2019). What is contentious is the role and function of simulation in supporting processes that were not traditionally thought to depend on a simulation. Representation and processing of conceptual content has been proposed to involve simulation of its corresponding perceptual, motor and interoceptive properties (Barsalou, 1999). The issue of boundary conditions for simulation has been intensely debated with respect to necessary vs. sufficient (or indeed epiphenomenal) contributions of simulation in conceptual processing.

The primary evidence typically cited for simulation is that sensorimotor regions/representations are almost immediately activated during tasks that “should” logically involve sensorimotor activity (e.g., picking, kicking, licking; Barsalou, 2016; Hauk, 2016; Hauk et al., 2004; Meteyard et al., 2012; Pulvermüller et al., 2005). Evidence *against* a simulationist interpretation comes from neuropsychological patients with acquired brain lesions who have demonstrated sensorimotor impairments but do not show the concomitant conceptual-level impairments that would be predicted by some versions of a simulationist approach (Mahon et al., 2009; Sartori et al., 2007). Despite entrenched views on these primary sources of evidence, the issues are complex. There are numerous sources of counterevidence for both views. Causal evidence for simulationist approaches (Möttönen & Watkins, 2009) and computational models show that modality-independent conceptual representations can arise in systems that are based on perceptual-motor simulation (L. Chen et al., 2017).

**Dissent #1 for simulation (Bedny):** There are two different notions of simulation used in the literature. The current definition focuses on the use of the term simulation form the embodiment perspective. However, simulations can also occur within abstract cognitive models. For example, intuitive theories of physics have been modeled as “physics engines” (Battaglia et al., 2013). Such models run simulations to predict the behavior of objects and substances. However, they need not refer to sensory or motor representations.

## General discussion

We produced a semantic glossary that included succinct definitions, background, agreement/confidence ratings, and principled dissents. We hope that this resource will provide a common ground (and nomenclature) for investigating semantic phenomena. The value of this glossary is not as a dogmatic set of definitions but as a reference point or snapshot in time for calibrating viewpoints across researchers and disciplines. Saying what we mean about semantic memory is a scientific best practice that will potentially improve construct specificity and promote incremental theory-building. Saying what we mean about semantic memory is also essential for falsification and unequivocal assessment of whether specified data or methods can support or refute a particular theory.

Construct specificity is an enduring challenge within both the physical and social sciences. The process of defining an unobservable construct is a human endeavor susceptible to human bias (Chang, 2009). The histories of science and medicine are rife with examples of theories (e.g., phlogiston, phrenology, luminous ether, miasma theory of disease) and therapeutic practices (e.g., whirling chairs for treating psychiatric disorders, trepanation for depression) that once enjoyed widespread acceptance but were later discredited. Structured consensus mechanisms provide formal countermeasures against many such biases (Linstone & Turoff, 1975).

Delphi methods typically rely on expert workgroups with the assumption that the forecast of a group (averaging across members) is generally more accurate than an individual’s prediction (see also the “wisdom of crowds”; Becker et al., 2017; Surowiecki, 2005). An illustrative example of a consensus process involves the establishment of formal diagnostic criteria for Alzheimer’s disease in 1984 based on the combined judgment of six investigators (McKhann et al., 1984). Neuroscience and medicine have since cultivated a deeper understanding of the pathology of Alzheimer’s disease and in turn have developed diagnostic tools (e.g., biomarker assays, radioligand imaging) that called for rethinking the diagnostic process. Revised consensus criteria were proposed in 2011 (McKhann et al., 2011), with more recent (and radical) biomarker-only diagnostic criteria in 2018 (Bradshaw & Georges, 2024; Jack et al., 2018). The evolution of diagnostic criteria for Alzheimer’s disease illustrates how a static snapshot today should be continuously re-evaluated, criticized, and reshaped by emerging scientific evidence tomorrow.

## Pitfalls of standardization and methodological limitations

Term *standardization* has many potential advantages for improving scientific rigor and reproducibility. However, such efforts have also caused harm when self-selecting groups of experts impose standards upon others. Delphi methods emphasize the importance of *representativeness* (Zartha Sossa et al., 2019). That is, composition of the workgroup should mirror the demographics and expertise of its intended audience. Although the community of semantic scholars is vast, we limited the size of the workgroup (*N*≫50) to facilitate meaningful interpersonal interactions among co-authors. Thus, this glossary only represents a tiny fraction of the semantic community.

Another limitation involved the stratified sampling procedure we used to identify expert contributors. We did not query gender identity, race, ethnicity, socioeconomic status, disability, religion, marital status, or any other personal information. In addition to constraints on workgroup size (*N*≫50) and a perfectly balanced sex ratio (1:1), selection criteria were informed by representation across: (1) Scientific disciplines (e.g., philosophy, linguistics, cognitive neuroscience); (2) Methodological expertise (e.g., neuroimaging, behavior); (3) Career stage (i.e., authors should span early to emeritus levels of experience); and (4) Geographic location of each expert’s faculty appointment (with attention to recruitment in southern hemisphere nations).

Although the overall composition of the workgroup satisfied many of these constraints, there is room for improvement. The workgroup had limited geographic and cross-cultural diversity. We recruited experts from countries where English is widely used and who publish extensively in English-language journals. The panel did not have extensive representation from semantic-adjacent disciplines (e.g., computer science, ethology, anthropology). In addition, we did not control for intersectionality. For example, several workgroup members were clinical neuropsychologists AND female AND L2 English speakers AND early career stage investigators. Each of these individual differences can pose significant challenges in conversational discourse, such as more intrusive interruptions (Anderson & Leaper, 1998) and lower perceived credibility (Lev-Ari & Keysar, 2010). Intersectionality of numerous individual differences can compound such biases, potentially silencing important voices. Of note, no panel member expressed concerns that their perspective had gone unheard or that they felt marginalized by our consensus process. Moreover, all dissenting co-authors endorsed disclosing their identities as a way of crediting and contextualizing their work. Yet lack of explicit concern does not preclude implicit bias.

One potentially sensitive indicator of bias involves a systematic pattern of individual differences among panel members who dissented (hereafter, dissenters). For example, if all dissenters were early career stage investigators, this might suggest an age or career stage bias in the consensus process. We conducted a post hoc audit of these factors with a focus on the potential for gender bias. Table 1 reflects relevant individual differences among the dissenters (*N* = 10).

**Table 1:** Dissenter demographics

Dissenter	N-Diss^1^	Sex	Career stage^2^	Geography	Discipline(s)
Bedny	4	F	Mid	USA	Psychology, Neuroscience
Bi	1	F	Mid	CHINA	Psychology, Neuroscience
Bolognesi	3	F	Early	ITALY	Linguistics
Fedorenko	3	F	Mid	USA	Linguistics, Neuroscience
Hoffman	1	M	Mid	UK	Psychology, Neuroscience
Lupyan	1	M	Senior	USA	Psychology, Neuroscience
Majid	5	F	Senior	UK	Psychology, Linguistics
Papeo	1	F	Early	FRANCE	Psychology, Neuroscience
Reilly	1	M	Mid	USA	Communication Disorders, Psychology
Yee	1	F	Mid	USA	Psychology

(1) N-Dissents reflects the raw total of dissents submitted by that particular panelist. (2) Career stage is a coarse distinction, especially among scientists who have completed years of postgraduate training. We adopted loose guidelines for early career as < 10 years of receiving terminal degree, mid-career as 10–20 years past the terminal degree, and senior as > 20 years. This distinction is entirely chronological rather than impact-based

There were no systematic differences in the distribution of dissenters as functions of age or scientific discipline. The sex distribution was skewed female (7:3), and several panelists submitted multiple dissents, skewing the raw dissent count to highly female (18:3). Another apparent difference was in the geographic distribution of the dissenters. All ten dissenters were from northern hemisphere nations. This distributional pattern must, however, be interpreted with caution because the base rate of scientists working in southern hemisphere nations was lower.

Why would someone feel the need to dissent from the majority position? One possibility is that the consensus process has chronically overlooked their perspectives. Alternatively, one might dissent repeatedly because they hold a constellation of views that differ from a canonical perspective. Finally, the presence of a dissent could indicate that a person feels empowered to express a difference of opinion that might otherwise be silenced by a standard consensus process premised upon anonymized majority voting. The critical point is that people might disagree with a majority position for many reasons. This is not to say that we employed unbiased processes but that ascribing motivation(s) for dissent comes with untenable assumptions that are not supported by the more positively disposed self-reports of the actual dissenters.

## Concluding remarks

The value of this glossary will be realized only if the broader semantic community recognizes its utility. These definitions will ideally serve as benchmarks for a larger debate about how the field might improve construct specificity and better embrace interdisciplinarity. Skepticism and criticism are healthy aspects of this process, and many more semantic constructs await characterization. We hope that others will join us in this discussion either in published commentaries or via a wiki produced for this purpose (https://consensussemantics.github.io/consensus_wiki).

## Data Availability

Not applicable.

## Footnotes

1 Tulving (1972) traced the earliest use of semantic memory to Paul Quillian’s doctoral dissertation (1966).

2 Semantic dementia (SD) was eventually reclassified as semantic variant primary progressive aphasia (svPPA) by a consensus workgroup (Gorno-Tempini et al., 2011). However, this shift in clinical terminology was not universally endorsed. Some researchers continue to reference semantic dementia. Co-existence of the terminology of SD and svPPA has created the uneasy impression that these disorders somehow represent two distinct clinical syndromes. This is another example of term ambiguity pervading medical diagnoses.

3 We discovered the unplanned necessary to account for historical drift in the meanings of numerous constructs. For example, Locke’s descriptions of abstractness in the late seventeenth century differ from modern conceptions of concreteness. Where applicable, we referenced historical and contemporary meanings of target constructs.

4 This definition has many similarities to the definition of concept earlier proposed by Malt and colleagues (2015).
